# New Process for the Sulfonation of Algal/PEI Biosorbent for Enhancing Sr(II) Removal from Aqueous Solutions—Application to Seawater

**DOI:** 10.3390/molecules27207128

**Published:** 2022-10-21

**Authors:** Mohammed F. Hamza, Eric Guibal, Khalid Althumayri, Thierry Vincent, Xiangbiao Yin, Yuezhou Wei, Wenlong Li

**Affiliations:** 1 School of Nuclear Science and Technology, University of South China, HengYang 421001, China; 2 Nuclear Materials Authority, P.O. Box 530, El-Maadi, Cairo 4710030, Egypt; 3 Polymers Composites and Hybrids, IMT—Mines Ales, F-30360 Ales, France; 4 Department of Chemistry, College of Science, Taibah University, Al-Madinah Al-Munawarah 30002, Saudi Arabia

**Keywords:** sulfonation of composite algal/PEI sorbent, strontium recovery, uptake kinetics and sorption isotherms, metal desorption and sorbent recycling, sorption selectivity, application to seawater, composite characterization, functionalization for enhanced performance

## Abstract

Sulfonic resins are highly efficient cation exchangers widely used for metal removal from aqueous solutions. Herein, a new sulfonation process is designed for the sulfonation of algal/PEI composite (A*PEI, by reaction with 2-propylene-1-sulfonic acid and hydroxylamine-O-sulfonic acid). The new sulfonated functionalized sorbent (SA*PEI) is successfully tested in batch systems for strontium recovery first in synthetic solutions before investigating with multi-component solutions and final validation with seawater samples. The chemical modification of A*PEI triples the sorption capacity for Sr(II) at pH 4 with a removal rate of up to 7% and 58% for A*PEI and SA*PEI, respectively (with SD: 0.67 g L^−1^). FTIR shows the strong contribution of sulfonate groups for the functionalized sorbent (in addition to amine and carboxylic groups from the support). The sorption is endothermic (increase in sorption with temperature). The sulfonation improves thermal stability and slightly enhances textural properties. This may explain the fast kinetics (which are controlled by the pseudo-first-order rate equation). The sulfonated sorbent shows a remarkable preference for Sr(II) over competitor mono-, di-, and tri-valent metal cations. Sorption properties are weakly influenced by the excess of NaCl; this can explain the outstanding sorption properties in the treatment of seawater samples. In addition, the sulfonated sorbent shows excellent stability at recycling (for at least 5 cycles), with a loss in capacity of around 2.2%. These preliminary results show the remarkable efficiency of the sorbent for Sr(II) removal from complex solutions (this could open perspectives for the treatment of contaminated seawater samples).

## 1. Introduction

Strontium is part of alkaline-earth metals; it is mainly extracted from celestite and strontianite minerals. Most of its uses are associated with optical and color-specific characteristics for fireworks (red-colored), glow-in-the-dark paints, and plastics (as Sr-aluminate). Strontium (as chloride salt) is also used in specialized toothpaste [1]. However, strontium mainly retains attention as a radioelement (^90^Sr) for its high-energy beta-emitter specificity. As a by-product of nuclear reaction and due to its similarities with calcium (meaning readily absorption and accumulation in bones and tissues), it remains a very hazardous compound for human and animal beings (as shown by the strong contamination of local seawater after Fukushima Daiichi disaster) [2,3,4,5,6].

These hazardous impacts have motivated for the last decades a strong research for elaborating new treatment processes. Metal removal may involve different techniques; however, sorption and biosorption processes have retained great attention for low-concentration effluents [7,8,9,10]. Though precipitation and flotation techniques can be used for pre-treating strontium-bearing effluents [11,12], it is generally useful to couple different techniques [13] for reaching high levels of metal recovery. For the specific treatment of complex solutions (such as brines and seawater), it is thus necessary to design new sorbents having high selectivity against alkaline and alkaline-earth competitor ions.

Inorganic sorbents have been widely investigated for strontium recovery using for example zeolites [14,15,16,17,18], or directly as nanostructured inorganic salts [19,20,21]. The association of ion-exchangers (IEs) with inorganic supports offers high selectivity for Sr(II) or Cs(I) associated with the cage effect of some IEs (such as Prussian blue, PB, and analogues) [22]. Similar concepts have been extended to PB immobilization onto carbon nanotubes [23], activated charcoal [24], polymer [25], or biopolymers [22,23,26]. Strontium removal was investigated using a wide range of bio-sorbents such as agriculture by-products [27], living microorganisms [28], yeast [29], and functionalized biopolymers [30,31]. Algal-based materials and sub-products have retained great attention for their potential to bind organic [32] and inorganic [9] contaminants.

Ion-exchange and chelating resins have been successfully tested for strontium binding from synthetic and for seawater solutions [33,34,35,36]. Functionalized polymers were designed as super-adsorbents for cesium and strontium, through the amidoximation of polyacrylonitrile/silica composites [37], or algal/PEI (polyethyleneimine) composite (A*PEI) [38]. Strong cation exchangers, such as sulfonic acid bearing resins (Dowex 50W series), have particularly retained attention for the sorption of strontium [16,39,40] or other solutes [41,42,43,44,45,46,47,48]. The efficiency of these sulfonated reactive groups for bearing strontium motivated the current research based on the functionalization of algal/PEI beads. Indeed, a new concept of composite support (obtained by the reaction of algal biomass with PEI with crosslinking and calcium ionotropic gelation, interpenetrated polymer network) was recently developed [49,50,51,52,53,54]. The high density of amine and hydroxyl groups in the composite offers many possibilities for functionalization by grafting specific reactive groups such as quaternary ammonium salt [51], amidoxime [38], and phosphoryl [53]. The sulfonation of algal/PEI beads revealed very efficient for improving the sorption properties for rare earth elements (i.e., Sc(III), Ce(III), and Ho(III) [55]). Actually, the grafting of new sulfonic groups brings not only additional functional groups with different affinities for target metal ions but also some complementary facilities associated with the bi-functionality of the sorbent (co-existing amine and sulfonic groups). Herein, a new process is designed for sulfonating algal/PEI beads (SA*PEI), based on the one-pot reaction of 2-propene-1-sulfonic acid and hydroxylamine-o-sulfonic acid with pristine A*PEI beads. This original method leads to the co-existence of different types of sulfonic groups.

In order to evaluate the potential of this sulfonic-functionalized sorbent, the materials are characterized using SEM coupled with EDX facilities, BET characterization, FTIR spectrometry, elemental analysis, and titration. The sorption properties are investigated through standard criteria such as the effect of pH, uptake kinetics, sorption isotherms, and competitive sorption (multi-component equimolar solutions). Metal desorption and sorbent recycling are studied and compared for A*PEI and SA*PEI. Strontium recovery is also characterized in very complex environmental such as seawater samples. The insights of this study count on: (a) the ecofriendly and cost-effective synthesis of a support based on alginate and algal biomass, (b) the production of a sorbent of highly physicochemical stability, (c) with outstanding efficiency for strontium removal (compared with alternative sorbents), and (d) fine selectivity from seawater treatment (based on the complementarity of reactive groups on the functionalized sorbent). Another merit of this work holds in the extensive characterization of the material and its interactions with strontium ions.

## 2. Results

### 2.1. Characterization of Materials

**Figure A1:**
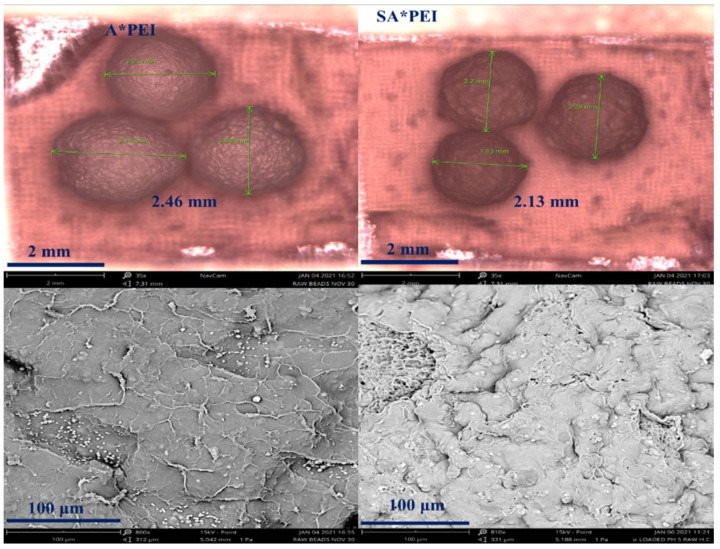
Optical microscope observations (**top**) and SEM observations (**bottom**) of A*PEI and SA*PEI sorbents.

**Figure A2:**
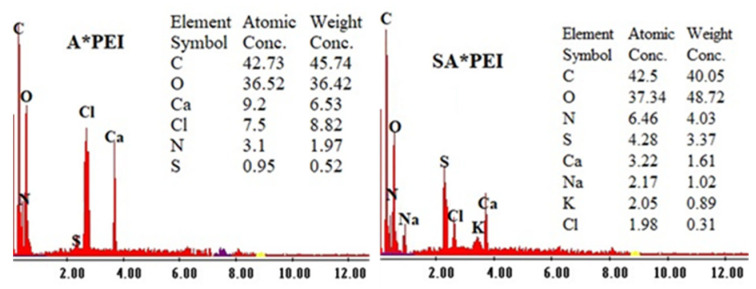
Semi-quantitative EDX analysis of the surface of A*PEI and SA*PEI sorbents.

The main characteristics are summarized below. A detailed discussion appears in Appendix A. The sorbents are roughly spherical (Figure A1) and their average sizes are 2.46 ± 0.21 mm and 2.14 ± 0.15 mm for A*PEI and SA*PEI, respectively. The sulfonation contributes to the weak shrinking of the beads. This is confirmed by the enlarged SEM observation of the surface of the sorbents: SA*PEI is characterized by a more wrinkled surface than A*PEI. The semi-quantitative EDX analysis of the surface of A*PEI and SA*PEI is reported in Figure A2. Substantial differences are observed considering the increases in N content (from 3.1% to 6.46%, At.%) and S content (from 0.95% to 4.28%, At.%). Notably, the functionalization of A*PEI leads to a significant decrease in Ca content (from 9.2% to 3.22%, At.%) while K and Na elements appear (up to 2.05% and 2.17%, At.%, respectively); concomitantly the atomic percentage of Cl element decreases from 7.5% to 1.98%.

**Figure A3:**
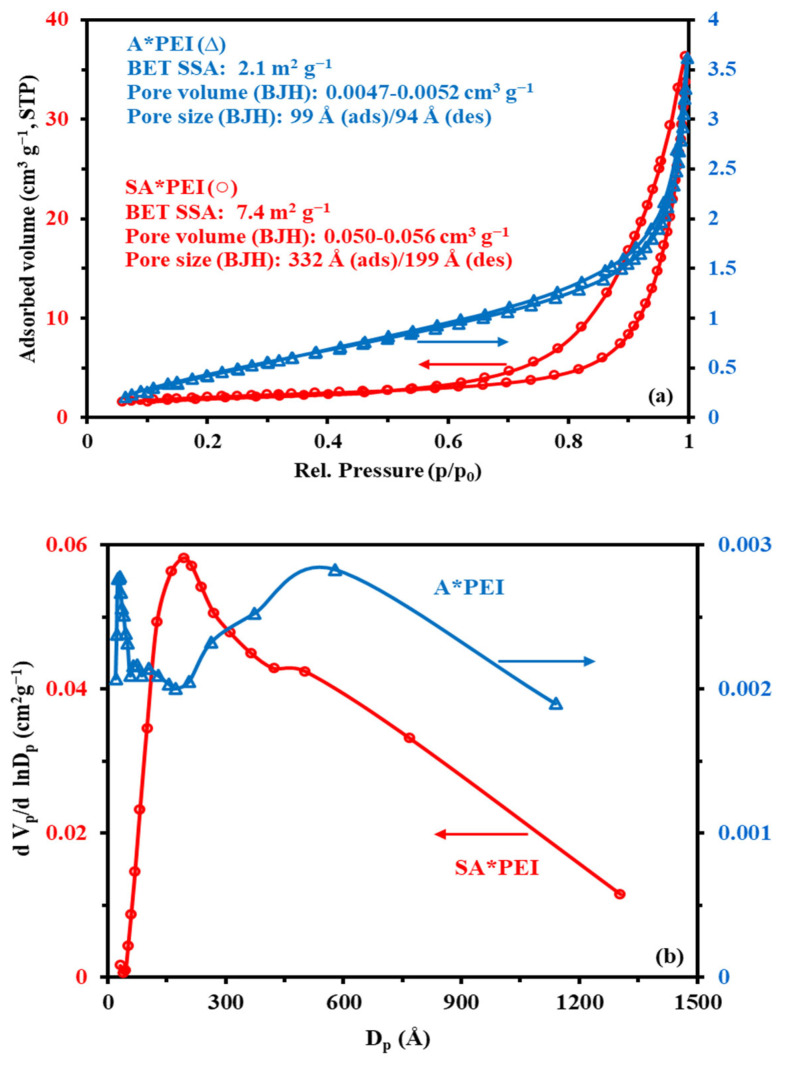
Textural properties of A*PEI and SA*APEI beads: (**a**) N_2_ adsorption/desorption isotherms, (**b**) pore size distribution.

The isotherms of N_2_ sorption and desorption show that the sulfonation of A*PEI increases the specific surface area of the pristine sorbent (from 2.1 to 7.4 m^2^ g^−1^) (Figure A3). The profiles show a substantial increase in the relative importance of the hysteresis loop. This functionalization also increases the porous volume (from ≈0.005 to 0.05 cm^3^ g^−1^), which is associated with a substantial increase in the pore width from 94–99 Å to 199–332 Å. Apparently, the chemical modification improves the ability of the sorbent for mass transfer. Despite low specific surface area (probably due to the large size of pores), the functionalized sorbent shows outstanding sorption properties for Sr(II) (see below).

**Figure 1:**
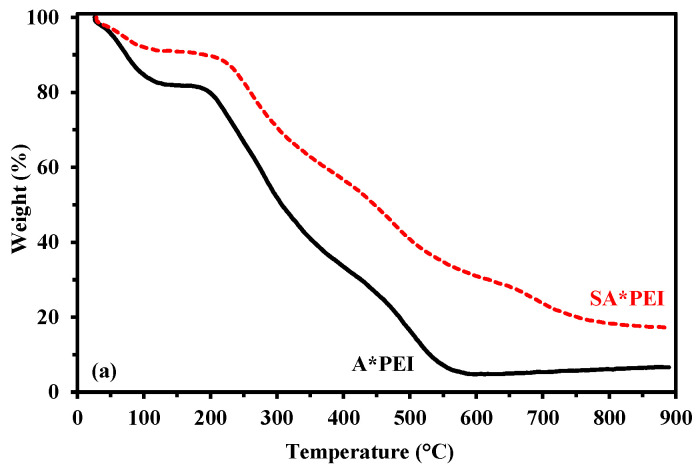

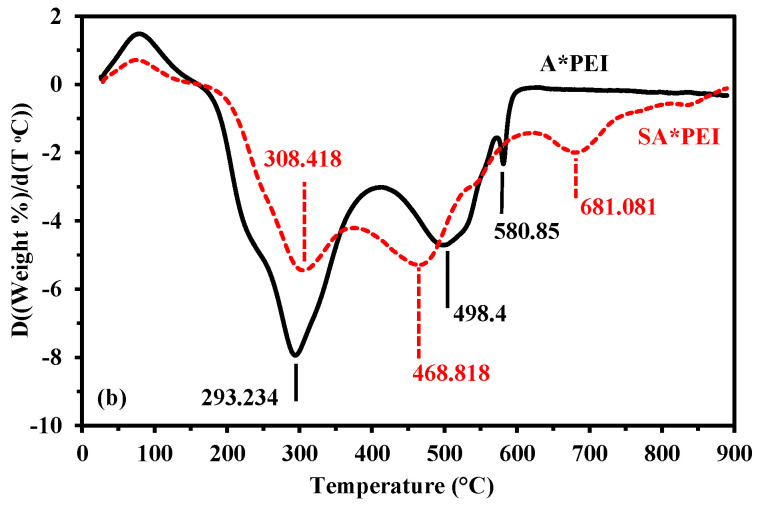
Thermal degradation of A*PEI and SA*PEI sorbents: (**a**) TGA, and (**b**) DrTG curves.

**Figure A4:**
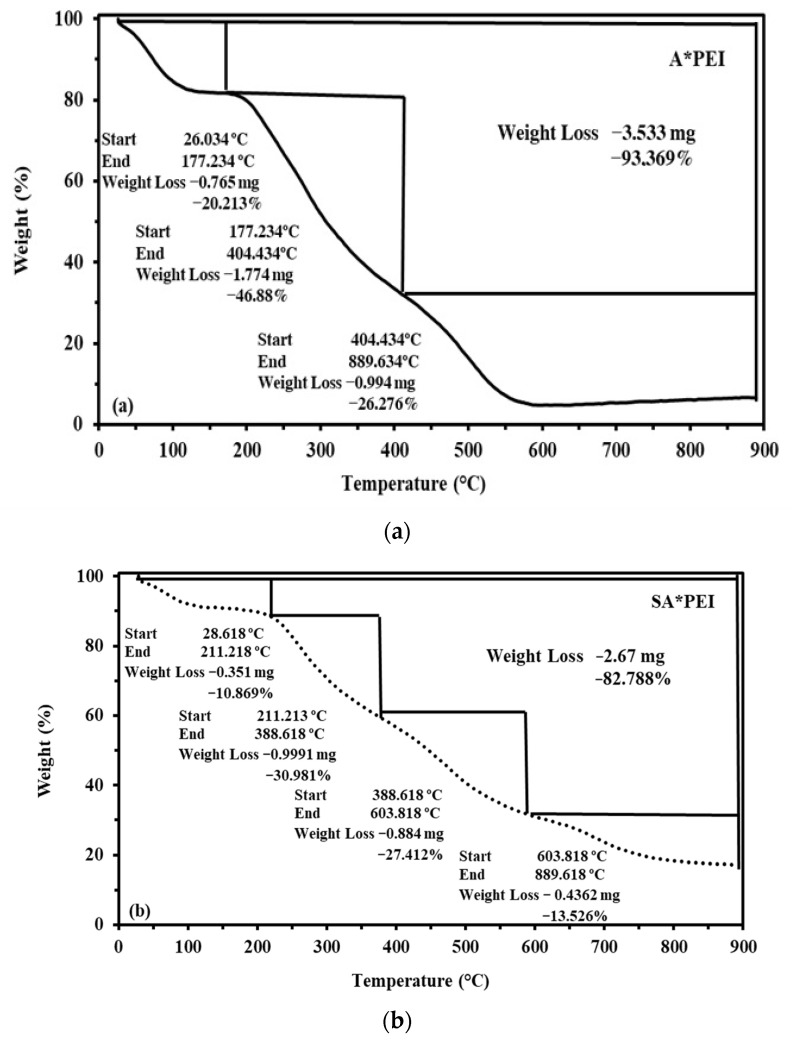
TGA analysis of A*PEI (**a**) and SA*PEI beads (**b**).

The thermal degradation also shows some differences in the shape of the TGA profiles (different weight-loss transitions) and some shifts in the specific temperatures (for DrTG profiles) (Figure 1). Apparently, the sulfonation increases the stability of the composite: Shifts in temperature and increased residue (about 17% vs. 7%). Apart from the same initial step (corresponding to water release, which represents different weight fractions), the profile for SA*PEI shows a supplementary transition compared with A*PEI, which may correspond to the specific degradation of sulfonate groups (Figure A4).

**Figure 2:**
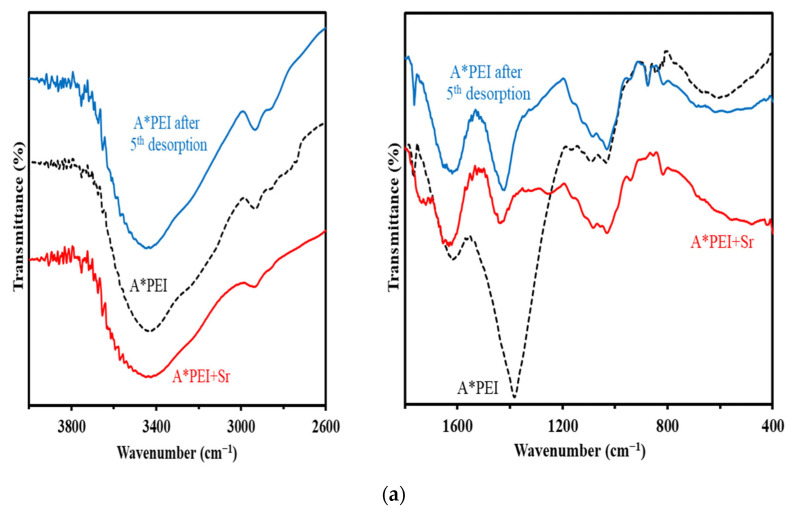

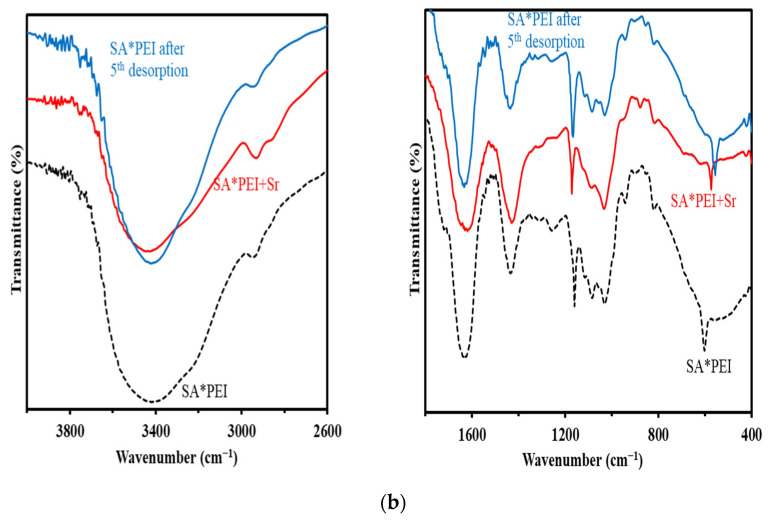
FTIR spectra of APEI (**a**) and SA*PEI (**b**) before and after Sr(II) sorption and after the fifth desorption (in recycling tests).

**Figure A5:**
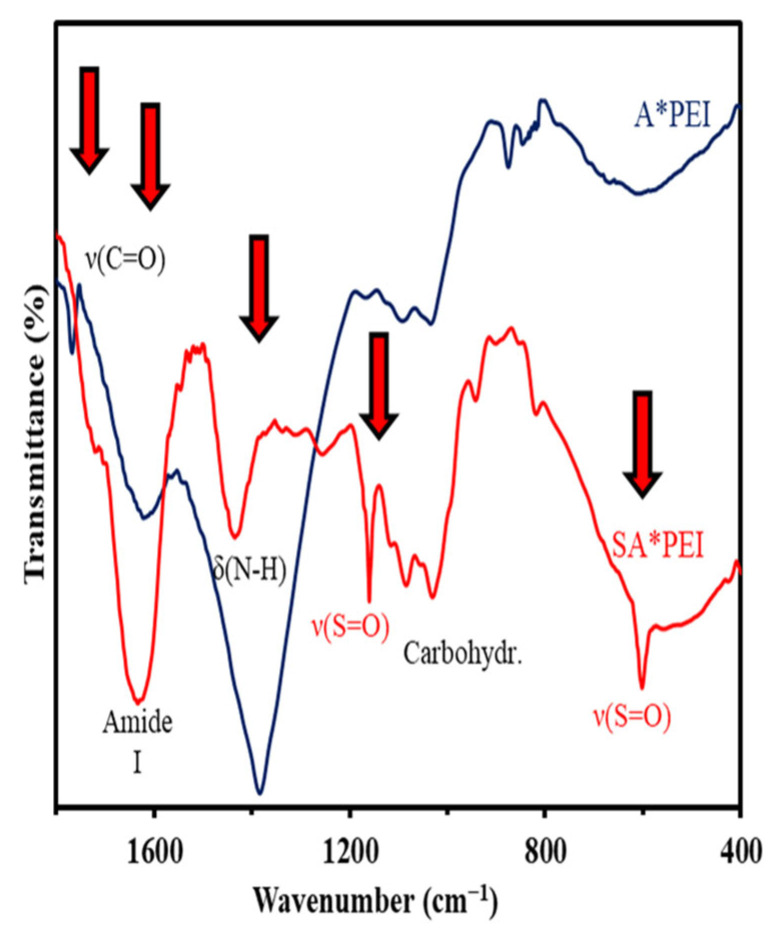
Main modifications of FTIR spectrum brought by the sulfonation of A*PEI.

The FTIR spectra for A*PEI and SA*PEI show: The presence of carbohydrate ring and more specifically uronic-based groups (mannuronic and guluronic acid moieties, 1300–1000 cm^−1^) and carbonyl from carboxyl groups (at 1767 cm^−1^) (Figure 2, Table A1). The interaction of alginate-based carboxylic acid with amine groups onto PEI is characterized by the formation of amide bonds (≈1620, ≈1400, and 1256 cm^−1^), which are partially superposed with amine bending vibration (Figure A5). The grafting of sulfonic acid is confirmed by the appearance (or reinforcement) of a series of bands at 1159, 850, and 602 cm^−1^.

**Figure A6:**
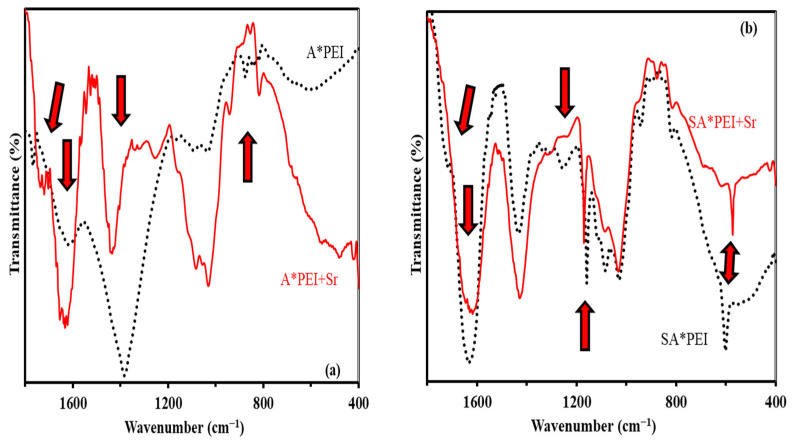
Main modifications of FTIR spectra of A*PEI (**a**) and SA*PEI (**b**) brought by Sr(II) sorption.

**Figure A7:**
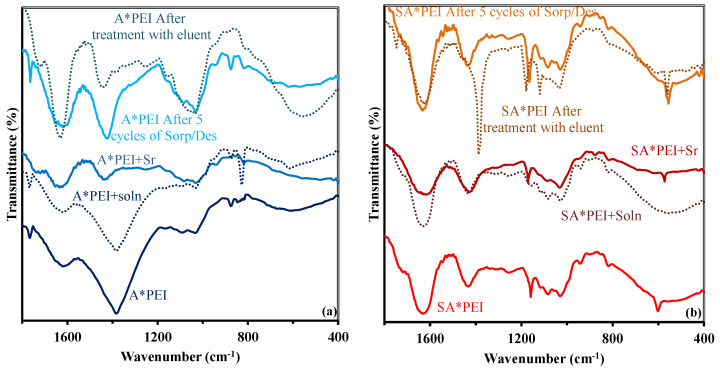
Effect of “environmental parameters” on the FTIR spectra of A*PEI (**a**) and SA*PEI (**b**) (sorbent after contact with solution at the pH of sorption, and sorbent after contact with the eluent).

The sorption of Sr(II) involves some changes in the FTIR spectra that may derive from the interactions of specific reactive groups with metal cations. Figure A6 isolates the main variations in FTIR spectra that confirm the interaction of Sr(II) with carboxylic and amine/amide groups in the case of A*PEI, while for SA*PEI, in addition, the chemical environment of sulfonate groups is also affected by metal binding. This binding is reversible (see Section 2.2.7), while using 0.3 M HCl solution as the eluent. As a consequence of metal desorption, the FTIR spectra are partially restored; however, the characteristic bands of reactive groups are not completely reestablished to their neat form (this may be the effect of protonation of reactive groups due to acidic elution) (Figure A7).

**Scheme 1:**
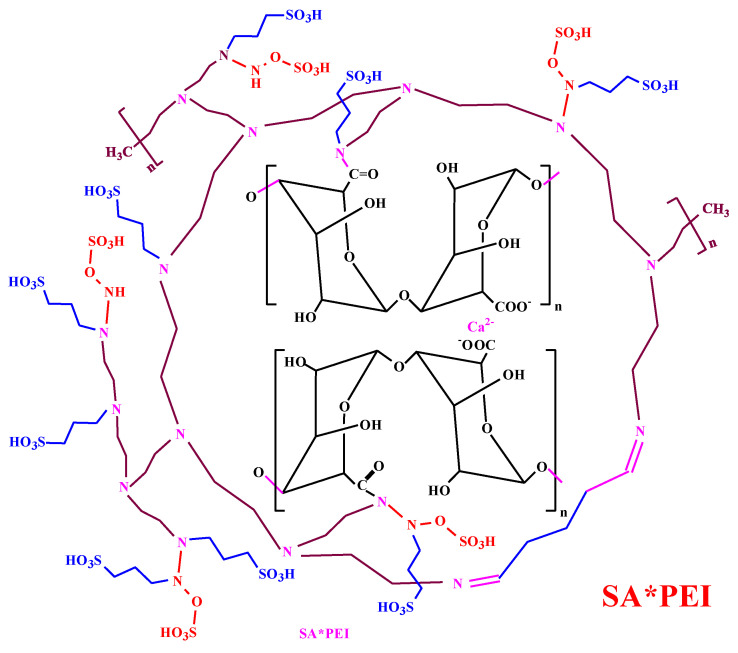
Structure of SA*PEI beads.

**Figure A8:**
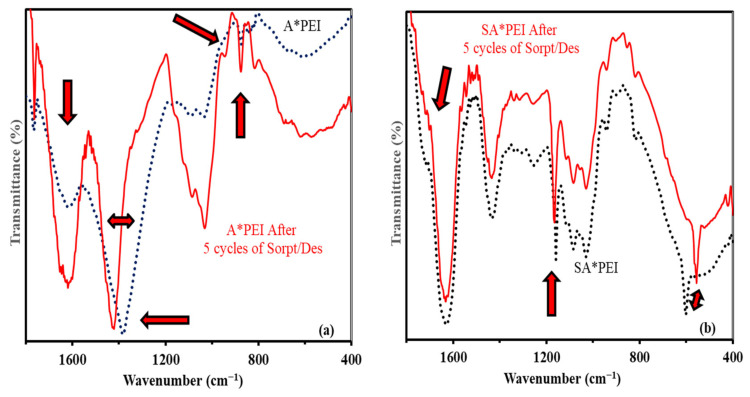
Main modifications of FTIR spectra of A*PEI (**a**) and SA*PEI (**b**) after the 5th cycle of sorption/desorption.

In Figure A7, the spectra of the materials at different stages of use are compared with the spectra of the sorbent exposed to solution (without strontium) at the pH of the sorption step and with the eluent solution (for metal-free sorbents). These spectra allow evaluation of the contributions of the environmental conditions and separate their effects on the spectra to specific changes associated with metal sorption and metal sorption/desorption cycles. The comparison of the spectra of pristine sorbents and those exposed to pH 5 solution (selected pH for strontium sorption) does not show significant differences (Figure A8): The changes in FTIR spectra after Sr(II) binding reported above can be specifically attributed to the interactions of functional groups with Sr^2+^. After conditioning the sorbent with 0.3 M HCl solutions (i.e., identical to the elution process for sorption/desorption cycles), the FTIR spectrum of A*PEI is more significantly changed at the level of carbonyl and amine groups (with stronger variations compared to the spectrum obtained after the 5 cycles). These functional groups are influenced by protonation and/or by metal binding to different extents). In the case of SA*PEI, the strong protonation affects the FTIR spectrum more significantly than successive cycles of sorption/desorption (the final rinsing step reduces the changes induced by protonation). In addition, the fine restoration of the FTIR profile confirms the good stability of the sorbent. Scheme 1 proposes the interaction modes of Sr(II) with functional groups held on A*PEI and SA*PEI. The grafting of 2-propene-1-sulfonic acid and hydroxylamine-o-sulfonic acid enriches the sulfonic and amine contents, which are involved in the binding of Sr^2+^ ions through chelation (i.e., hydroxyl and amine) and ion-exchange (through replacement of Ca^2+^ with Sr^2+^ and the protons from sulfonic groups). The functionalization opens to a wider variety of sorption mechanisms and a larger number of reactive groups than the pristine sorbent.

The elemental analysis of the two sorbents confirms the effective grafting of sulfonic groups (reaching ≈ 1.04 mmol S g^−1^) (Table A2). Based on the structure of grafted moieties and the elemental analysis, the density of sulfonate groups is close to 1 mmol g^−1^. Nitrogen content remains unchanged (≈2.14 mmol N g^−1^), while O content increases by 4.68 mmol O g^−1^ after the functionalization of A*PEI. In 2-propene-1-sulfonic acid (2P1SA) and hydroxylamine-O-sulfonic acid (HOSA), the increase in O content is 3 and 4 times the S content. The relative decrease of A*PEI fraction in the sulfonated derivative is compensated by hydroxylamine-O-sulfonic acid grafting. It is noteworthy that the increase in O content exceeds the expected stoichiometric ratio O/S in the insertion of sulfonic derivatives: The ratio reaches 4.52:1, while the ratio would be expected between 3:1 (for 2P1SA) and 4 (for HOSA). This may be due to traces of reagents; semi-quantitative EDX analysis shows traces of K^+^ and Na^+^ from potassium persulfate and sodium bisulfite; different levels of hydration (water absorption) may also explain theses discrepancies.

**Figure A9:**
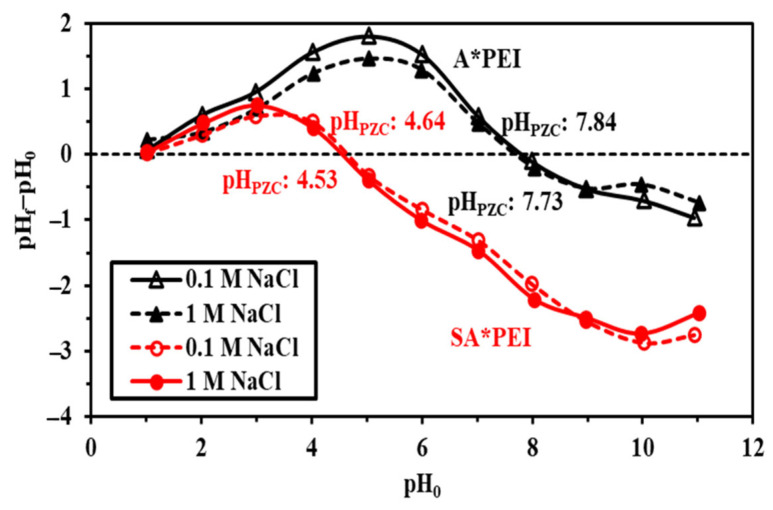
pH_PZC_ determination using pH-drift method for A*PEI and SA*APEI (sorbent dose, SD: 2 g L^−1^; background salt: NaCl (at concentrations 0.1 M and 1 M); time: 48 h).

Figure A9 reports the evaluation of pH_PZC_ values for the two sorbents: Data were collected from two sets of experiments using different concentrations of background salt. The profiles hardly changed. The grafting of sulfonic acid onto amine groups (of PEI) logically gives a stronger acid character to the sorbent: The pH_PZC_ is shifted from ≈7.8 to ≈4.6. In the case of sulfonation of similar algal/PEI beads, Hamza et al. [55] reported a stronger decrease in pH_PZC_ values (from 7.35 to 2.86); in the previous work, sulfosuccinic acid was used for the functionalization of the neat sorbent. Obviously, these acid–base characteristics strongly affect the surface charge of the sorbent: The global charge is positive in acidic solutions for SA*PEI (below pH 4.6) while it is necessary to increase the pH to 7.8 for losing the cationic charge in the case of A*PEI. This may have a direct impact on the attraction/repulsion of divalent cations such as Sr^2+^.

### 2.2. Sorption Properties—Synthetic Solutions

#### 2.2.1. pH Effect on Sr(II) Sorption

**Figure 3:**
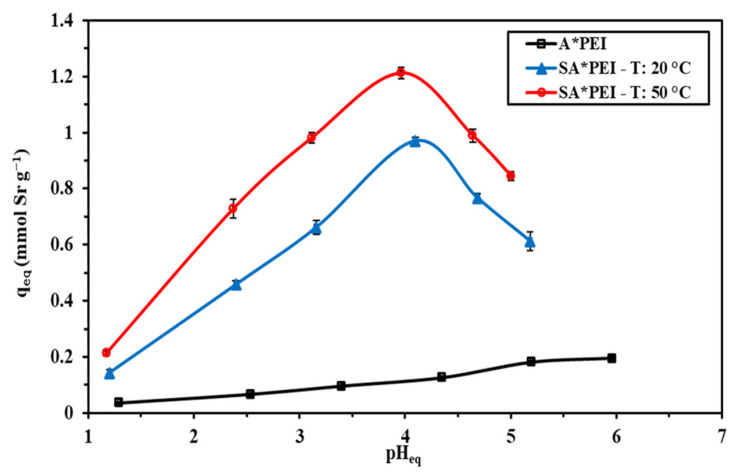
pH effect on Sr(II) sorption using A*PEI and SA*PEI beads—sorption capacity vs. pH_eq_ (C_0_: 1.17 mmol Sr L^−1^; Sorbent dose, SD: 0.666 g L^−1^; T: 20 ± 1 °C (except for SA*PEI test at T: 50 ± 1 °C); time: 48 h; averaged values and standard deviations).

**Figure A10:**
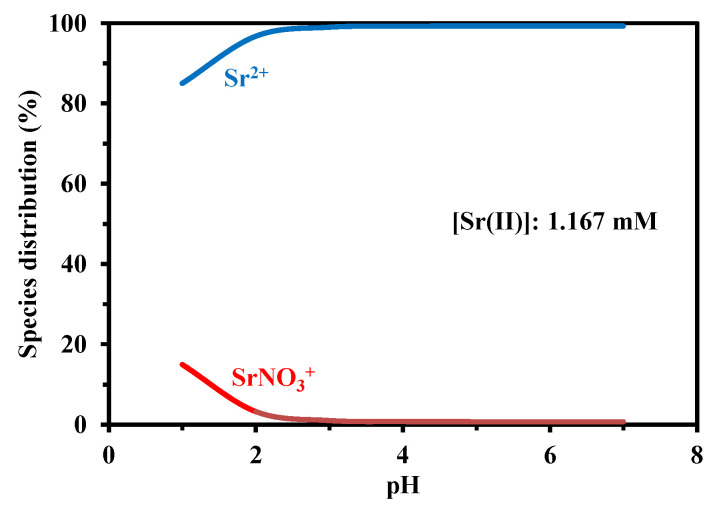
pH effect on Sr(II) speciation (under experimental conditions from pH study; calculated using Visual Minteq, [108]).

Figure 3 shows the comparison of the average values (triplicated series) of the pH-profiles for the sorption of Sr(II) using A*PEI and SA*PEI. Under selected experimental conditions (C_0_: 1.17 mmol Sr L^−1^, and sorbent dosage, SD: 0.67 g L^−1^), the sorption capacity linearly increases with pH_eq_ (up to pH 6) but remains quite low; never exceeding 0.21 mmol Sr g^−1^. The beneficial effect of sulfonation is clearly demonstrated: The sorption capacity strongly increases for SA*PEI from 0.122 to 0.98 mmol Sr g^−1^, when pH_eq_ increases from 1.2 to 4.1. Above pH_eq_ 4, the sorption capacity sharply decreases (down to 0.6 mmol Sr g^−1^ at pH_eq_ 5.2). The effect of pH may be expressed by two main reasons associated with (a) the change in the speciation of the metal (charge of the solute, formation of complexes including polynuclear species or colloids, depending on the metal), and/or (b) the change in the charge of the sorbent (protonation or deprotonation of reactive groups). Under selected conditions, the speciation diagram shows in Figure A10 that strontium is only present as free species (i.e., Sr^2+^) above pH 3, while below pH 3 a small fraction is present as SrNO_3_^+^. Therefore, strontium remains cationic and poorly affected in the pH range investigated herein; the strong impact of pH on sorption capacity is not driven by the speciation characteristics. In acidic solutions, the strong protonation of reactive groups (especially for A*PEI) limits metal cation sorption and it is necessary to increase the pH for enabling Sr^2+^ binding. The main functional groups present on A*PEI are carboxylic acid (alginate fraction of algal biomass) and amino groups (from PEI). PEI bears primary, secondary, and tertiary amino groups with pK_a_ values close to 4.5, 6.7, and 11.6, respectively [56], while mannuronic and guluronic constituents of alginate have pK_a_ values close to 3.38 and 3.65, respectively [57]. In acidic solutions, most of the amine groups remain protonated repulsing metal cations. On the opposite side, carboxylic groups progressively deprotonate (mannuronic before guluronic acid) and carboxylate groups may bind strontium by electrostatic attraction or ion-exchange with calcium ions bound to carboxylate groups. Hong et al. [58] also reported that the sorption of Sr(II) onto alginate beads increases with pH and tends to stabilize at a pH higher than 4.

The sulfonation of A*PEI strongly changes the pH-profile with maximum sorption close to pH 4. Sulfonic acids are strong acids; the negative apparent pK_a_ values vary with the type of substituent (as shown by Dong et al. [59] for benzenesulfonic derivatives). This means that anionic sulfonate groups may coexist on the sorbent with weakly acidic carboxylic groups and alkaline amine groups; the pH_PZC_ is close to 4.6: The global charge of the sorbent remains positive in acidic conditions and progressively decrease. Strontium cations can bind first to sulfonate groups before carboxylate groups begin to contribute with increasing the pH. The competition of protons with Sr^2+^ cations can also explain the weak sorption in strongly acidic pH regions. Surprisingly, the sorption capacity steeply decreases above pH_eq_ 4, despite the favorable conditions for sorbent charge (and simultaneous deprotonation of sulfonic, carboxylic, and primary amine groups). The semi-quantitative analysis of the sorbent surface (using EDX) showed the presence of Na^+^ and K^+^, completed by the increase of sodium content due to pH control; this may explain a shielding effect at the surface of the material. This effect limits the ability of negatively-charged functional groups to bind strontium cations under slightly alkaline conditions. It is noteworthy that the same experiment was performed at increased temperature (i.e., T: 50 °C), the pH-edge curve shows the same trends: A sharp optimum is found again close to pH_eq_ 4. However, the curve is shifted toward higher sorption capacities: The sorption of Sr(II) onto SA*PEI is endothermic.

**Figure A11:**
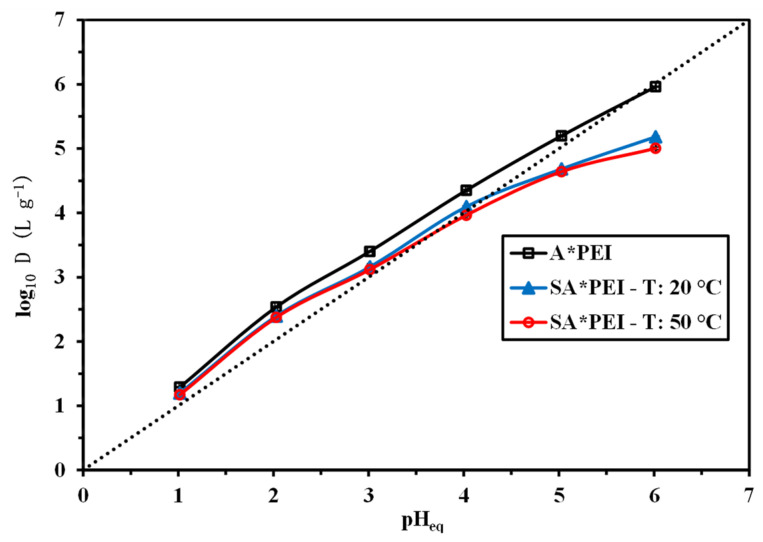
pH effect on Sr(II) sorption using A*PEI and SA*PEI beads—pH_eq_ vs. pH_0_ (C_0_: 1.17 mmol Sr L^−1^; Sorbent dosage, SD: 0.666 g L^−1^; T: 20 ± 1 °C (except for SA*PEI test at T: 50 ± 1 °C); time: 48 h).

**Figure A12:**
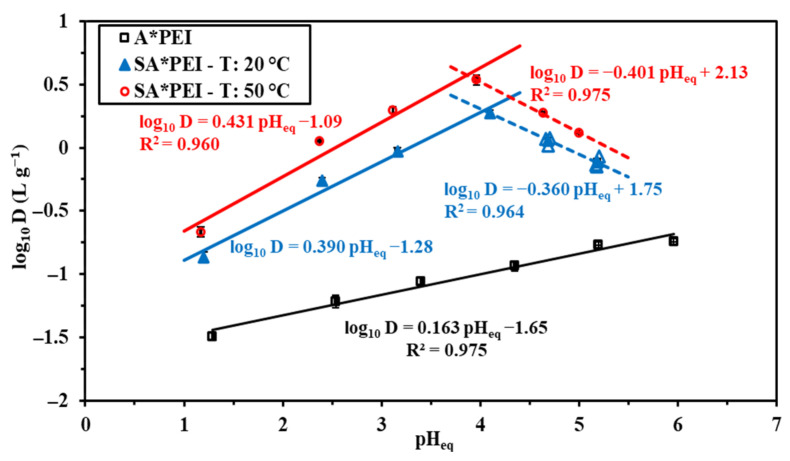
pH effect on Sr(II) sorption using A*PEI and SA*PEI beads—distribution ratio vs. pH_eq_ (C_0_: 1.17 mmol Sr L^−1^; SD: 0.666 g L^−1^; T: 20 ± 1 °C (except for SA*PEI test at T: 50 ± 1 °C); time: 48 h).

Figure A11 compares initial and equilibrium pH values after strontium sorption. For A*PEI, the pH tends to weakly increase (especially at pH close to 2, by 0.37–0.55 pH unit): Protons are bound; at pH 6, the equilibrium pH remains stable. The sulfonation affects the pH change: The final pH increases by less than 0.4 pH unit between pH 1 and pH 3, the pH remains unchanged up to pH 4 and tends to decrease at pH above 4 (associated with proton release dye ion-exchange with Sr(II) binding. This breakpoint is close to the pH_PZC_ value of functionalized beads. In Figure A12, the log_10_ plots of the distribution ratio (D = q_eq_/C_eq_, L g^−1^) vs. pH_eq_ show a good correlation but the slopes cannot be clearly associated with conventional ion-exchange ratio (see Appendix B.2).

At acidic pH (i.e., pH 1–2), the protonation of hydroxyl groups (from algal backbone in A*PEI) and both amine and sulfonic groups (in SA*PEI). Under these conditions, Sr(II) is bound through an ion-exchange mechanism. With the increase of the pH, the reactive groups progressively deprotonate and some reactive groups become negatively-charged making possible the binding of Sr(II) ion through a binary mechanism of ion-exchange and chelation. Associated with the decrease of ionic repulsion, the sorption of Sr(II) is enhanced. At pH above the pH_PZC_ value, the sorption capacity for SA*PEI strongly decreases probably due to the shielding and competition effects of Na^+^, K^+^, and Ca^2+^ (as appearing in semi-quantitative EDX analysis) and in relation to the progressive increase in the negatively-charged surface of the sorbent (sulfonate groups). Similar phenomena were reported for the sorption of U(VI) using two chelating resins [60], and for the development of superabsorbent polymers [61,62]. The screening of anionic charges with Na^+^ affects the configuration and packing of the chains but also contributes to the decrease in the availability of reactive groups. This decrease is not observed for A*PEI sorbent, where binding is limited to free amine and carboxylic groups. Further experiments were performed at optimum pH conditions: pH_0_ 4 for SA*PEI and pH_0_ 5 for A*PEI.

#### 2.2.2. Uptake Kinetics

**Figure 4:**
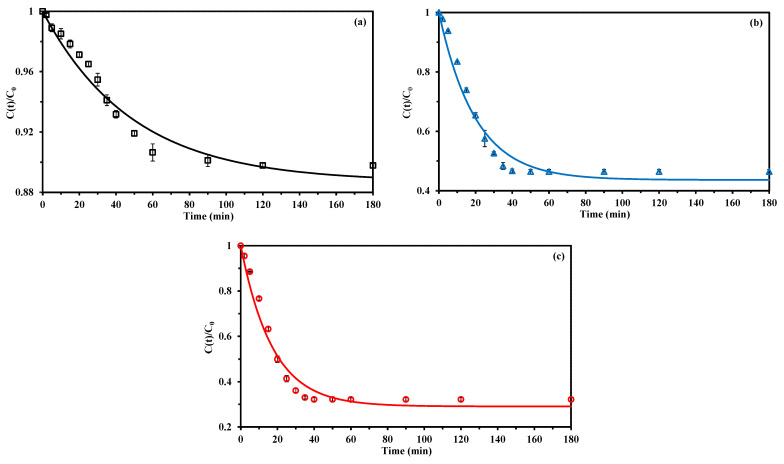
Sr(II) uptake kinetics for A*PEI (**a**) and SA*PEI (**b**,**c**) beads—Modeling with PFORE (C_0_: 1.17 mmol Sr L^−1^; SD: 0.666 g L^−1^; T: 20 ± 1 °C (**a**,**b**) and at T: 50 ± 1 °C (for SA*PEI, c); pH_0_: 5 and 4 for A*PEI and SA*PEI, resp.; averaged values/std. dev.).

The kinetic profiles for Sr(II) sorption using A*PEI and SA*PEI are compared in Figure 4 (under the same experimental conditions, except pH values adjusted to their optimal values). For A*PEI, 90–120 min are necessary for reaching the equilibrium; apparently, there is a break in the slope of the concentration decay at around 30–35 min, possibly associated with the hydration of the sorbent. The kinetics is faster and more homogeneous in the case of SA*PEI: The equilibrium time is reduced to 30–40 min. this faster mass transfer may be correlated with the textural characteristics of the sorbents: SA*PEI has a slightly greater specific surface area and larger pores than the precursor (i.e., A*PEI). The sorption kinetics are controlled by mechanisms of resistance to diffusion (essentially film and intraparticle diffusions) and by the reaction rate (which can be described by pseudo-first, pseudo-second-order rate equations; PFORE and PSORE, Table A3 (a)). The resistance to film diffusion was negligible (due to the appropriate pre-selection of stirring speed); in addition, the resistance to film diffusion is also minimized by the appropriate choice of agitation conditions (herein: 210 rpm). This mechanism is mainly active in the overall control of uptake kinetics within the first minutes of contact. The initial section corresponds, under these conditions, to the fitting of the experimental profile with a first-order rate equation. With a proper agitation rate, the resistances to bulk diffusion and film diffusion can be neglected.

Table 1 summarizes the apparent rate coefficients for PFORE and PSORE (k_1_ and k_2_, respectively); the statistical parameters are also compiled for the resistance to intraparticle diffusion (i.e., RIDE, Table A3 (a)). Table A4 reports the parameters and statistical data for individual replicates.

The comparison of the determination coefficient (R^2^) and Akaike Information Coefficient (AIC) clearly demonstrates that the PFORE best fits experimental profiles than the PSORE (and even better the RIDE). In the literature, the mathematical preference for a given model is frequently used for discriminating between physical and chemical sorption; however, this interpretation requires some conditions to be fulfilled (such as a negligible variation of the sorbate concentration in the solution), which are rarely verified; making the interpretation questionable [63]. Herein, except for A*PEI (for which the weak sorption limits concentration change), the conditions are not respected for appropriate interpretation. However, the parameters are useful for comparing the sorbents and evaluating the effect of temperature (in the case of SA*PEI). The solid lines in Figure 4 represent the simulated profiles with PFORE: triplicated curves confirm the good reproducibility of sorption tests. It is noteworthy that increasing the temperature slightly increases the sorption kinetics and the sorption capacity (consistently with the observation reported in the study of the pH effect). The faster sorption of Sr(II) by SA*PEI is confirmed by the ranging of apparent rate coefficients (k_1_, min^−1^) according to:

A*PEI (2.05 ± 0.13 × 10^−2^ min^−1^) < SA*PEI (5.02 ± 0.23 × 10^−2^ min^−1^).

These values are of the same order of magnitude as the apparent rate coefficients reported for Dowex 50W-X8 (i.e., 0.0214 min^−1^, [36]) but higher than for surfactant-conditioned polyacrylonitrile (i.e., 0.0022 min^−1^, [64]) or for biochar and magnetic biochar (i.e., 0.0047 and 0.0077 min^−1^, respectively, [65]). Bezhin et al. [66] reported much lower values for Sr(II) sorption kinetics using a series of resins and ion-exchangers (i.e., in the range 0.0015–0.0024 min^−1^). It is noteworthy that in most of these studies the PSORE gave better fitting of kinetic curves than the PFORE. The calculated values of sorption capacities at equilibrium general overestimate the experimental results (see Appendix B.3).

The intraparticle diffusivity coefficient (deduced from the application of the RIDE to experimental profiles) hardly varies with the sorbent: 6.84 ± 0.22 × 10^−9^ m^2^ min^−1^ for A*PEI and 7.2–8.1 × 10^−9^ m^2^ min^−1^ for SA*PEI (depending on the temperature). This means that the intraparticle transfer is little enhanced into the slightly more porous sorbent (i.e., SA*PEI). These values are comparable to the diffusivity coefficient (i.e., 7.2 × 10^−9^ m^2^ min^−1^) reported by Morig and Gopala Rao [67] in the case of Dowex 50W-X8 sulfonic resin. These values are only one order of magnitude lower than the self-diffusivity of Sr(II) in water (i.e., 4.76 × 10^−8^ m^2^ min^−1^, [68]). Strontium diffusion in A*PEI and SA*PEI sorbents is facilitated compared with porous polymer-coated hydroxyapatite (i.e., 1.8 × 10^−10^ m^2^ min^−1^) [69].

#### 2.2.3. Sorption Isotherms

**Figure 5:**
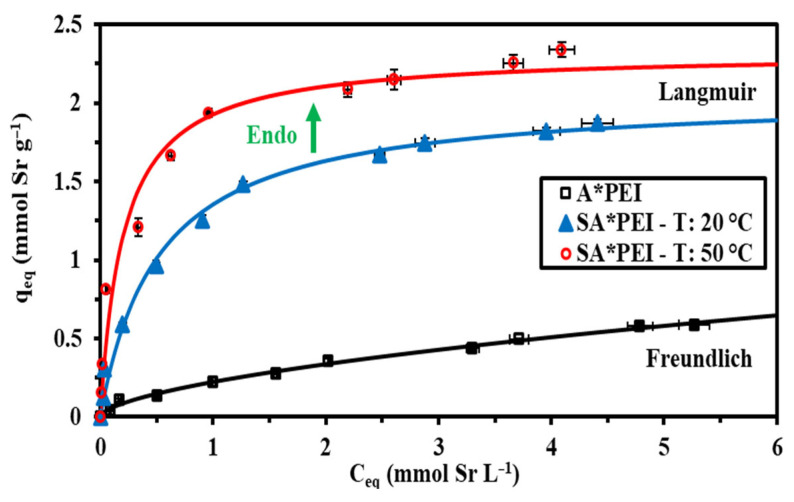
Sr(II) sorption isotherms on A*PEI and SA*PEI beads—Modeling with Langmuir equation or Freundlich equation (C_0_: 0.12–5.82 mmol Sr L^−1^; SD: 0.666 g L^−1^; T: 20 ± 1 °C (for A*PEI and SA*APEI) and at T: 50 ± 1 °C (for SA*PEI, c); pH_0_: 5 and 4 for A*PEI and SA*PEI, resp.; average values/std.dev.; fitting on cumulated series).

Sorption isotherms are summarized in Figure 5 (q_eq_ vs. C_eq_); the triplicate series are superposed: The sorption tests are reproducible. Consistently with previous results, the sulfonation of A*PEI strongly enhances the sorption of strontium: Both (a) the saturation plateau (i.e., the maximum sorption capacity for C_eq_ ≈ 5 mmol Sr g^−1^) from 0.587 ± 0.015 to 1.870 ± 0.029 mmol Sr g^−1^, and (b) the affinity coefficient (which is correlated with the initial slope of the curve). In A*PEI, carboxylate and amine groups potentially contribute to Sr(II) binding with low affinity: The sorption capacity weakly progresses with increasing metal concentration. Based on the nominal values of their pK_a_, the deprotonation of the reactive groups makes carboxylate groups more available than primary amine groups present on PEI for Sr(II) binding. The sulfonation brings additional reactive groups that are good cation exchangers and remain fully deprotonated at pH 4. These strong reactive groups may explain the sharp increase in sorption capacity: The initial slope is much steeper (stronger affinity) and the density of reactive groups increases.

Figure 5 also presents the sorption isotherm at T: 50 °C for SA*PEI. Both the maximum sorption capacity (from 1.870 ± 0.029 to 2.340 ± 0.049 mmol Sr g^−1^) and the affinity increase with sorbent functionalization: This is consistent with the results obtained in the study of pH effect and uptake kinetics. Increasing the temperature enhances metal binding; the sorption of Sr(II) onto SA*PEI is endothermic. This probably means that the mechanism of Sr(II) binding to sulfonate groups is endothermic. Wang et al. [70] modified the macroporous styrene chelating resin (with diglycolamidic acid functional group) by grafting sulfonic groups for Pb(II) binding: They found that metal sorption is endothermic. Shin et al. [65] also reported endothermic sorption of Sr(II) onto biochar (and magnetic biochar). A similar conclusion was obtained in the case of Sr(II) binding onto sulfactant-functionalized polyacrylonitrile [64].

The sorption isotherms were fitted with different models (appearing in Table A3 (b)). In Figure 5, the solid lines represent the fitting of isotherms for SA*PEI with the Langmuir equation at pH_0_ 4 (and the Freundlich equation for A*PEI at pH_0_ 5). The parameters of the different models are reported in Table 2 (combining the triplicated series; Table A5 (a–c) report the individualized treatment of the experimental series). Maximum sorption at saturation of the monolayer (Langmuir model) substantially increases after functionalization (from 0.997 ± 0.113 to 2.057 ± 0.052 mmol Sr g^−1^), as does also the affinity coefficient (b_L_: From 0.282 ± 0.070 to 1.943 ± 0.164 L mmol^−1^). With the endothermic nature of Sr(II) sorption onto SA*PEI, the Langmuir parameters increase at T: 50 °C up to 2.327 ± 0.045 mmol Sr g^−1^ and 4.760 ± 0.099 L mmol^−1^.

**Figure A13:**
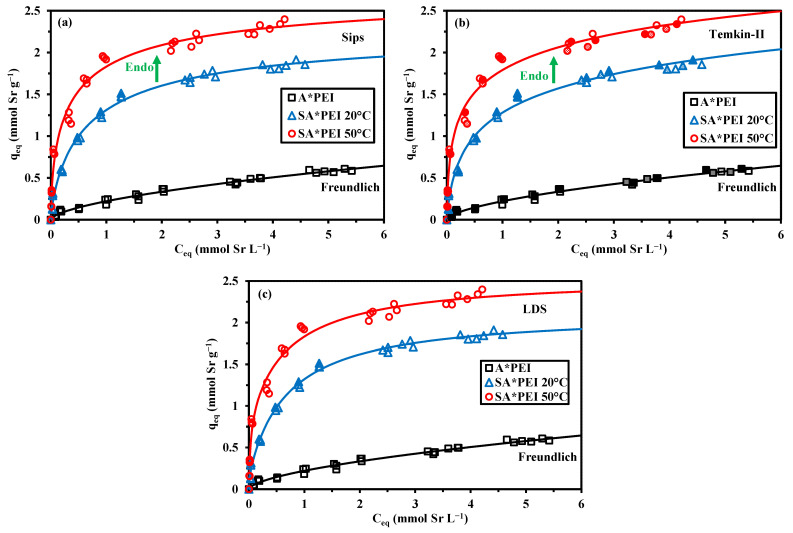
Sr(II) sorption isotherms on A*PEI and SA*PEI beads—Modeling with Freundlich equation for A*PEI and Sips equation (**a**), Temkin-II equation (**b**), and Langmuir dual site (LDS) (**c**) for SA*PEI (C_0_: 0.12–5.82 mmol Sr L^−1^; SD: 0.666 g L^−1^; T: 20 ± 1 °C (for A*PEI and SA*APEI) and at T: 50 ± 1 °C (for SA*PEI, c); pH_0_: 5; triplicated series; fitting on cumulated series).

Alternate fittings are presented in Figure A13. The comparison of the different models shows that for A*PEI sorbent the Freundlich equation fits better experimental data than the Langmuir and the Sips equations. This model supposes that sorption occurs with possible interactions between sorbed molecules and with heterogeneous energies of sorption. On the opposite hand, for SA*PEI sorbent the Sips equation (with a combination of Langmuir and Freundlich equations) is more appropriate. The Langmuir equation is a mechanistic equation based on the homogeneous sorption of sorbate at the surface of the sorbent, as a monolayer without interactions between sorbate molecules. The Sips equation introduces a third-adjustable parameter that may contribute to better mathematical fit (at the expense of a loss in physical significance) (Figure A13a). The Temkin equation assumes that the heat of adsorption of the molecules bound onto the monolayer linearly decreases with its relative coverage and that there is a uniform distribution of heterogeneous binding sites. It is usually inappropriate to fit the two extreme regions of the isotherm (i.e., low concentration and saturation zone) [71]. This may explain the lower quality of curve fitting (with the remarkable exception of the isotherm at 50 °C for SA*PEI. Chu [71] reported and corrected the dimensional inconsistency commonly found in publications applying the Temkin equation to solid/liquid sorption. In addition, several derived equations one of these forms allows accommodating the Temkin equation to low concentration ranges (called Temkin-II equation (Equation (1)), by opposition to the conventional equation reported in Table 2):

(1) $$\mathrm{Temkin-II}\;q_{\mathrm{eq}}={\;q}_{T}\;\ln\left(1+K_{T}{\;C}_{\mathrm{eq}}\right)$$

Herein, the Temkin-II equation improves the quality of the fit for SA*PEI at T: 20 °C; while the improvement for T: 50 °C is less significant (Figure A13b).

The functionalized sorbent bears sulfonate groups in additional to the reactive groups present on A*PEI. These reactive groups may have different affinities for the target metal; this introduces heterogeneities at the surface of the sorbent, which can be accounted for using the Langmuir dual site equation (LDS) [72]:

(2) $$q_{\mathrm{eq}}=\frac{q_{L1}{\;b}_{L1}{\;C}_{\mathrm{eq}}}{1+b_{L1}{\;C}_{\mathrm{eq}}}+\frac{q_{L2}{\;b}_{L2}{\;C}_{\mathrm{eq}}}{1+b_{L2}{\;C}_{\mathrm{eq}}}$$

with q_Li_ (mmol g^−1^), b_Li_ (L mmol^−1^) as the maximum sorption capacities, and Langmuir affinity coefficients for sites i = 1 and 2.

**Figure A14:**
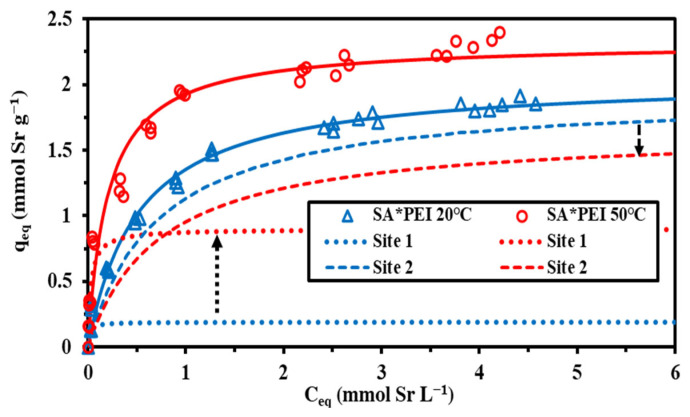
Contributions of Sites 1 and 2 in the Sr(II) sorption isotherms for SA*PEI at T: 20 and 50 °C.

The LDS equation fits well experimental profiles (Figure A13c and Table 2, lowest values of the AIC). One of the reactive groups is characterized by high sorption capacity with low affinity (Site 1), while the other has a strong affinity for Sr(II) but with lower density (sorption capacity) (Site 2). It is noteworthy that increasing the temperature differently affects these two reactive groups: For Site 2, the parameters are weakly decreased at t: 50 °C, while for Site 1, the maximum sorption capacity significantly increases but the affinity is halved. Figure A14 compares the contributions of Sites 1 and 2 at 20 and 50 °C. With increasing temperature, the relative contribution of Site 1 substantially increases (from ≈10% at T: 20 °C to 39% at T: 50 °C) [72].

Table A6 reports Sr(II) sorption performances of a series of alternative sorbents. *Bacillus pumilus* bacteria show outstanding sorption capacity (about 3.4 mmol Sr g^−1^) under environmental conditions (i.e., at pH 7) with weak affinity and slow kinetics (equilibrium requires about five days of contact) [28]. Similarly, granular manganese oxide shows relatively good sorption capacity, very weak affinity, and long equilibrium time [73]. The sorption properties of SA*PEI, based on the compromise between sorption capacity and affinity, kinetics, and pH, are comparable to those reported for amidoximated algal/PEI beads [38], functionalized silica beads [74], functionalized graphene oxide [75], and graphene oxide [76]. Compared with a commercial ion-exchanger (i.e., sulfonated Dowex 50W-X8 resin) that also bears sulfonic groups, the sorption capacity, determined at lower pH (i.e., 3.7), is of the same order (as well as the equilibrium time) while the affinity coefficient is significantly higher (up to 206 L mmol^−1^). Based on the selected criteria, SA*PEI is part of the most efficient sorbents for Sr(II), as an alternative to Dowex 50W-X8 resin.

#### 2.2.4. Binding Mechanisms

**Scheme 2:**
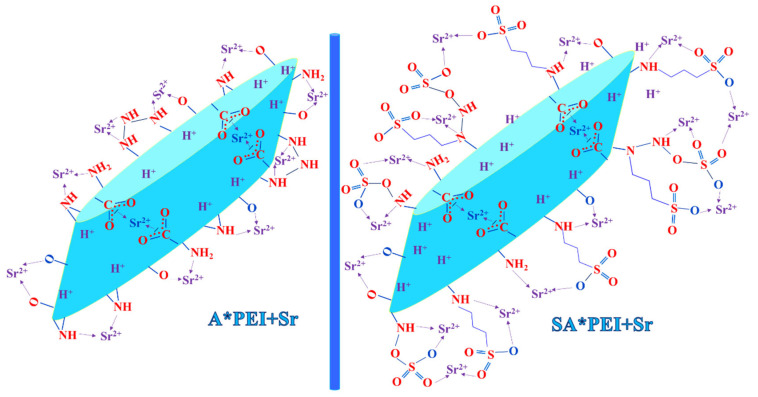
Suggested mechanism of Sr(II) interactions with A*PEI and SA*PEI.

Elemental analysis, titration, and FTIR characterization have shown the effective sulfonation of the pristine sorbent. The comparison of sorption properties of A*PEI and SA*PEI showed a strong improvement in sorption properties. This is due to the supplementary binding of Sr(II) to the oxygen-based moieties of sulfonic groups; this is characterized by the decrease in the relative intensities of –OH and sulfone groups bands in FTIR characterization after metal binding. The chelation of strontium cations (related to metal speciation) onto –OH and amine groups results from a favorable balance between the charge of the sorbent (resulting from pH_PZC_ characterization and the study of pH effect on metal binding) and the metal charge. On the other side, Ca^2+^ ions may be replaced with Sr^2+^ through an ion-exchange mechanism. Scheme 2 shows the expected mechanism for the sorption of Sr(II) ions onto pristine beads (A*PEI) and the sulfonated sorbent (SA*PEI).

#### 2.2.5. Sorption Selectivity

The sorption of Sr(II) onto SA*PEI may be influenced by the presence of other metal ions which may have a certain affinity for the reactive groups present on the multi-functional sorbent (carboxylic, amine and sulfonic groups). Obviously, the competition effect exerted by their presence depends on the pH (tough the cross effects of protonation/deprotonation of reactive groups and metal speciation). In order to evaluate this potential effect, the sorption capacity of Sr(II) is determined at different pH values in multi-component equimolar solutions containing Na(I) alkaline element), Ca(II) and Mg(II) (alkaline-earth elements), Fe(III), and Al(III) (heavy metal elements). Under these conditions, it is meaningful and easy to calculate the selectivity coefficient for Sr(II) against other metal ions (i.e., SC_Sr/metal_; ratio of relevant distribution ratios) as:

(3) $${\mathrm{SC}}_{\mathrm{Sr}/\mathrm{metal}}=\frac{D_{\mathrm{Sr}}}{D_{\mathrm{metal}}}=\frac{q_{\mathrm{eq},\mathrm{Sr}}\times {\;C}_{\mathrm{eq},\mathrm{metal}}}{C_{\mathrm{eq},\mathrm{Sr}}\times {\;q}_{\mathrm{eq},\mathrm{metal}}}$$

**Figure 6:**
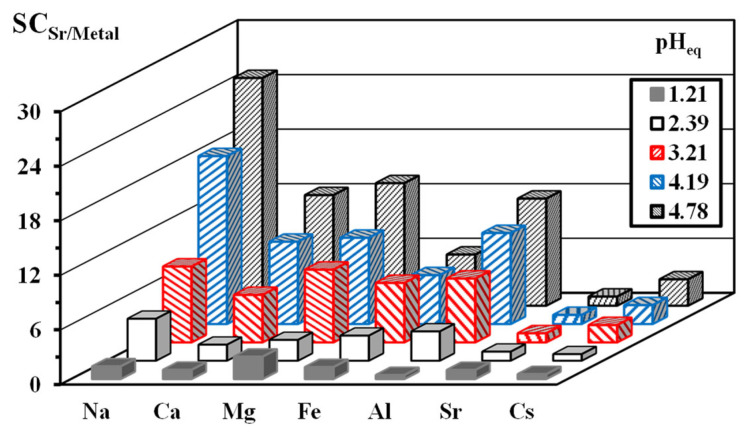
Effect of pH on the selectivity coefficient SC_Sr/Metal_ for multi-component equimolar solutions (C_0_: 1 mmol L^−1^; SD: 1.6 g L^−1^; T: 20 ± 1 °C; time: 24 h).

Figure 6 shows that the selectivity of SA*PEI for Sr(II) against competitor metal ions increases with the pH of the solution (for Fe(III) almost equivalent between pH_eq_ 3.21 and 4.78). At pH_eq_ 4.78, the selectivity ranking for SC_Sr/metal_ follows the order:

Cs(I) (2.9) > Fe(III) (5.6) > Al(III) (11.8) > Ca(II) (12.2) > Mg(II) (13.5) > Na(I) (25.0).

**Figure A15:**
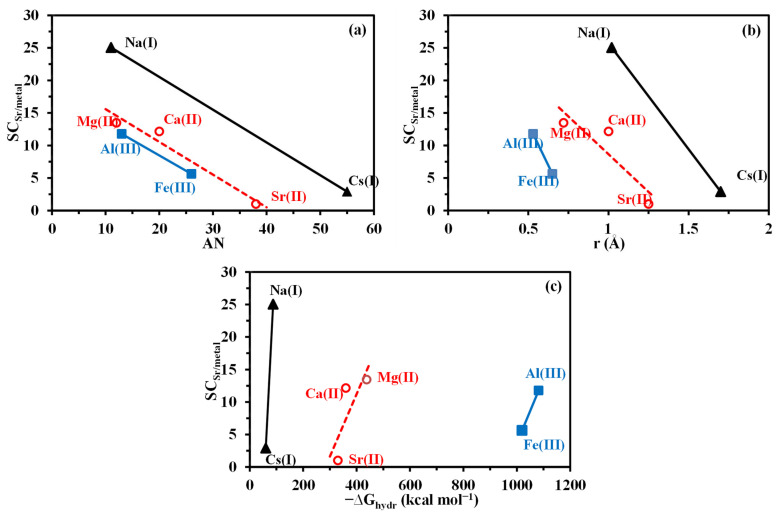
Correlation of SC_Sr/metal_ with Atomic Number (AN) (**a**), ionic radius (**b**) and enthalpy of hydration (**c**) (grouped by ionic charge: +1, +2, and +3).

In the case of Dowex 50W-X8 sulfonic resin, Bonner [77] reported the selectivity of the resin according: Cs(I) > Na(I) and Sr(II) > Ca(II) > Mg(II) (also confirmed for divalent cations by Iyer et al. [78]). This is roughly consistent with the specific observation collected herein. Pelhivan and Altun [79] established that the selectivity of sulfonic functions on Dowex 50W increases with atomic number, valence, and degree of ionization of exchanged metal ions. On the other side, Gupta [80] reported the effect of hydration enthalpy of monovalent ions. These different correlations are reported in Figure A15. The data are grouped by ionic charge and show that in each family the selectivity coefficient for Sr(II) against competitor ions: (a) decreases with increasing the atomic number, and the ionic radius and (b) increases with the hydration enthalpy (absolute value). Comparing the competitor effects of Ca(II) and Mg(II) with that of K(I) on Sr(II) sorption by resorcinol-formaldehyde resin, Nur et al. [34] highlighted the effect of charge difference and the inner-sphere complexation behavior of divalent cations.

**Figure A16:**
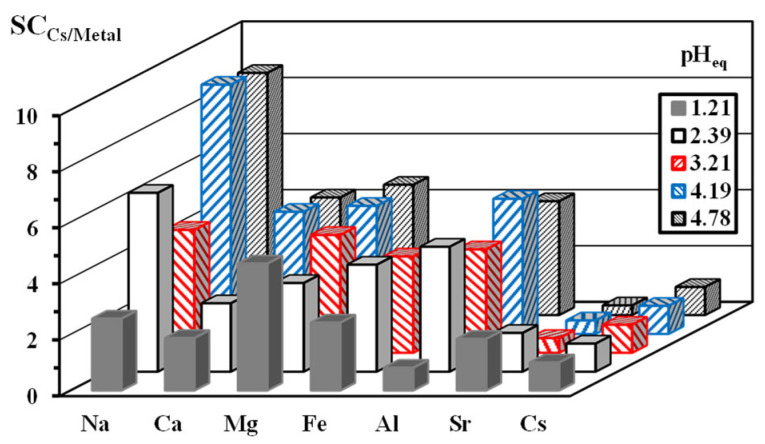
Effect of pH on the selectivity coefficient SC_Cs/Metal_ for multi-component equimolar solutions (C_0_: 1 mmol L^−1^; SD: 1.6 g L^−1^; T: 20 ± 1 °C; time: 24 h).

The sorbent has clearly a marked preference for Sr(II) and Cs(I). Figure A16 shows the SC_Cs/metal_ for the different metals with varying pH. The response to pH is less “monotonous” than for Sr(II) selectivity. Hence, the optimum separation of Cs(I) from Sr(II), Fe(III) occurs at pH_eq_ 1.21 and 2.39, respectively; while for other metal ions, the highest values for SC are reported at pH_eq_ 4.19–4.78.

**Figure A17:**
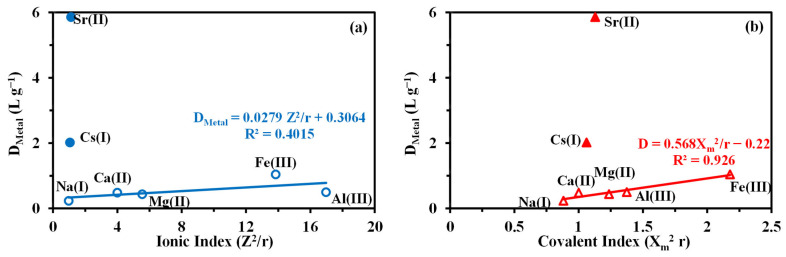
Distribution ratios vs. Ionic Index (Z^2^/r) (**a**) and covalent index (X_m_^2^ r) (**b**).

Figure A17 tests the correlation of the affinity of the metals for SA*PEI with their physicochemical properties: The individual distribution coefficients are plotted against the ionic index (i.e., I.I. = Z^2^/r) and the covalent index (i.e., C.I. = X_m_^2^ r), where Z and X_m_ are the charge and the Pauling electronegativity of metal ions. Figure A17 clearly confirms the out-of-trend behavior of SA*PEI for Sr(II) and Cs(I) compared with the other competitor ions: Their distribution ratio is much larger for Sr(II) > Cs(I) and much more for the other mono-, di-, tri-valent cations. For the competitor ions, the distribution ratio follows a linear trend against C.I (higher correlation than for I.I.).

#### 2.2.6. Effect of Salinity (NaCl) on Sr(II) Sorption

**Figure A18:**
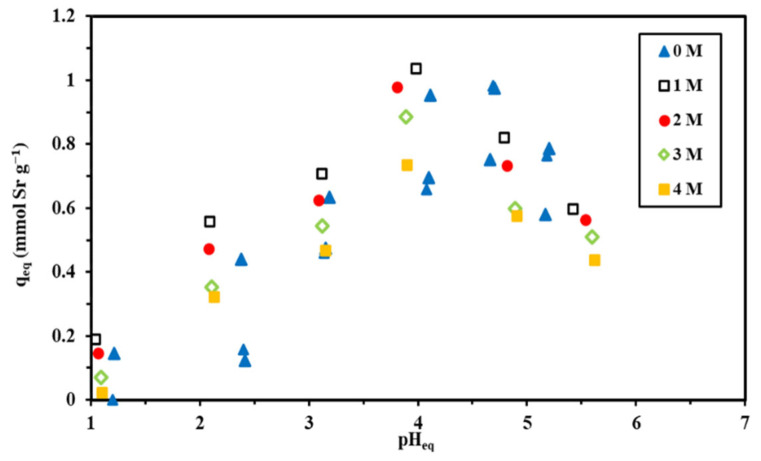
Influence of NaCl concentration on Sr(II) sorption (C_0_: 1.17 mmol Sr L^−1^; SD: 1 g L^−1^; T: 20 ± 1 °C; time: 20 h; Curve 0 M correspond to Figure 2 and SD: 0.666 g L^−1^; time 48 h; average value/std. dev. for 3 replicates).

In an attempt to evaluate the potential of SA*PEI to be applied for seawater treatment, the effect of NaCl concentration is investigated: Figure A18 compares the effect of pH on Sr(II) capacity with increasing Na,Cl concentrations (from 1 to 4 M; the curve without NaCl addition is reported as a reminder, though processed under different experimental conditions). The salt concentration does not change the global behavior of the sorbent: The sorption capacity increases up to pH_eq_ 4 before decreasing. The slopes of the two sections are parallel and hardly changed by salt concentration. The decrease in sorption capacity becomes significant above 2 M. However, even with a Na,Cl concentration as high as 4 M, the sorption capacity, at pH_eq_ ≈4, only decreases by 26% (from 0.996 mmol Sr g^−1^, average value for 0–2 M data, vs. 0.735 mmol Sr g^−1^). The little decrease in sorption capacities may be explained by the co-sorption of Na(I) (not shown, up to 20 mmol Na g^−1^ at the highest Na,Cl concentration and higher pH value); however, despite this strong sorption, the decrease is relatively limited, meaning that different functional groups are involved in Na(I) and Sr(II) binding. The mineral charge in seawater does not exceed 3.5% (w/w); with Na,Cl content close to 0.65 M. Despite an excess of NaCl as high as 2 M (even higher than in seawater), the sorption of Sr(II) is hardly affected: Sorption capacity remains close to 0.97 mmol Sr g^−1^ (under selected experimental conditions). This stability is remarkable because of the risk for alginate to be partially dissolved in a such high concentration of sodium (ion-exchange of Ca^2+^ with Na^+^); this means that the interpenetrating network (alginate/PEI with ionotropic gelation and glutaraldehyde crosslinking) contributes to stabilizing the material. This outstanding stability in sorption performance in complex solutions contrasts with the significant decrease in strontium sorption observed by Yin et al. [81], while using biogene-derived aerogels (imprinted dopamine-alginate materials): The sorption efficiency was divided by three even with Na,Cl concentration as low as 0.1 M. In the case of alginate microsphere, the sorption capacity of Sr(II) was decreased by 60% in presence of NaCl (0.65 M) [58]. In the presence of high concentrations of NaCl, Ca-alginate may be converted into Na-alginate, which can be dissolved and involve the de-structuration of the hydrogel. However, apparently under concentrations as high as 3–4 M NaCl, the multiple mechanisms of crosslinking involved in the preparation of the sorbent (alginate interaction with amine groups (at appropriate pH), amine crosslinking with glutaraldehyde, and ionotropic gelation of carboxylate groups with Ca(II) lead to stable interpenetrating networks. Therefore, the reduction in sorption performance at the highest concentrations may be due to a partial degradation of the hydrogel; however, the material shows a good overall stability.

The distribution ratios (i.e., D, L g^−1^, not shown) increase with pH for Na(I), contrary to Sr(II) (which showed a maximum for pH 4), and decrease with increasing Na,Cl concentration (from 0.0093 to 0.0029 L g^−1^). At pH 4, the D ratio for Sr(II) is 215 to 465 times higher than the value for Na(I). This is consistent with competitor studies (Section 2.2.5 and Section 2.2.6) and with the remarkable preference of SA*PEI for Sr(II) against Na(I); this is a good indication of the interest of the sorbent for application in seawater (see Section 2.3).

#### 2.2.7. Sr(II) Desorption from Metal-Loaded SA*PEI and Sorbent Recycling

Strontium desorption was operated using an acidic solution; the weak sorption in acidic solutions is the first incentive for using acidic solutions. Hong et al. [58] reported the successful desorption of strontium from alginate beads; however, they pointed out substantial degradation at repeated use. Therefore, they used 0.1 M HCl/CaCl_2_ solutions; the presence of calcium allows for re-structuring of the hydrogel beads by ionotropic gelation. A combined 0.2 M HCl/0.5 M CaCl_2_ solution was used for recovering strontium from amidoximated algal/PEI beads, with complete desorption of the metal and stable re-use. In the case of granular manganese oxide, strontium was recovered with 1 M HNO_3_ solution as the eluent [73]. They obtained very low desorption rates with this sorbent where the metal is tightly bound.

**Figure A19:**
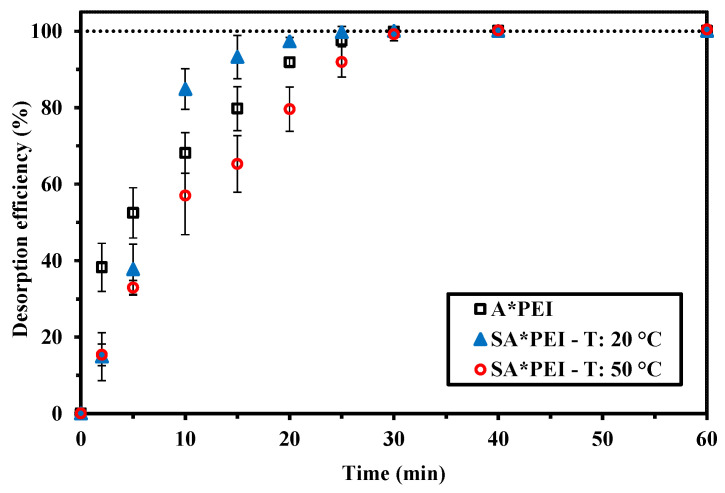
Desorption kinetics for Sr(II)-loaded sorbents (collected from uptake kinetics) using 0.3 M HCl solutions (SD: 2.67 g L^−1^; T: 20 ± 1 °C; v: 210 rpm).

Figure A19 compares the kinetic profiles for Sr(II) desorption using the metal-loaded materials recovered at the end of uptake kinetics and 0.3 M HCl solution as the eluent. Some discrepancies are observed in the repetition tests; however, in all cases, the desorption is fully achieved within 20–40 min. Apparently, the desorption is slightly faster for SA*PEI compared with A*PEI at 20 °C. It is noteworthy that the slowest desorption kinetics are obtained for the sorbent loaded at 50 °C: The thermal activation leads to a stronger interaction between the sorbent and Sr(II) (consistently with the affinity coefficients).

The re-use of the sorbents (over 5 cycles) is reported in Table 3. The two sorbents show remarkable stability in terms of desorption efficiency along the five cycles. However, the comparison of sorption efficiencies shows marked differences between A*PEI and SA*PEI. In the case of A*PEI, the sorption progressively decreases during the recycling tests. Since the desorption is complete, the loss in sorption capacity, which reaches up to 12% in the fifth cycle, is probably associated with a degradation of the sorbent. Some changes have been observed in the FTIR analysis (see Section 2.1), as a confirmation of this hypothesis. On the opposite hand, the sulfonated material shows outstanding stability in sorption efficiencies (the loss at the fifth cycle is less than 2%).

Different types of eluent have been reported for strontium elution, for example, sodium carbonate in the case of imprinted bio-hydrogel (with a loss in sorption that reaches up to 17% at the fifth cycle) [81]. In the case of analogous sulfonic resin (Dowex 50W-X8), Hafizi et al. [36] reported that EDTA is more efficient than acid solutions for Sr(II) elution; however, the recycling of the sorbent was not qualified in their contribution.

Note: Complementary interpretations and analyses of experimental data on sorption properties are reported in Appendix B.

### 2.3. Application to Complex Solution—Seawater

Section 2.2.5 confirmed that SA*PEI has a marked preference for Sr(II) against a series of alkaline, alkaline-earth, and heavy metals (and Cs(I) to a lesser extent). The next step consists in testing the sorption performances in even more complex systems such as seawater. Table A7 reports the composition of two samples collected from the Mediterranean and the Red Sea coasts. Strontium is present in concentrations as low as 4–6 mg Sr L^−1^ with a very large excess of Na(I) (up to 14 g Na L^−1^), Mg(II) (up to 1.5 g Mg L^−1^). As a corollary, chloride ions are in huge excess (about 19 g Cl L^−1^). These tests are necessary for evaluating the potential of this material for the treatment of contaminated seawater (such as resulting from the Fukushima Daiichi disaster, [82]).

**Figure A20:**
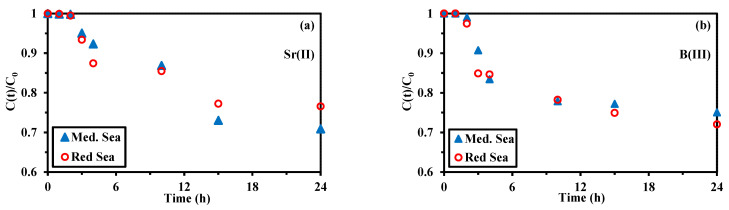

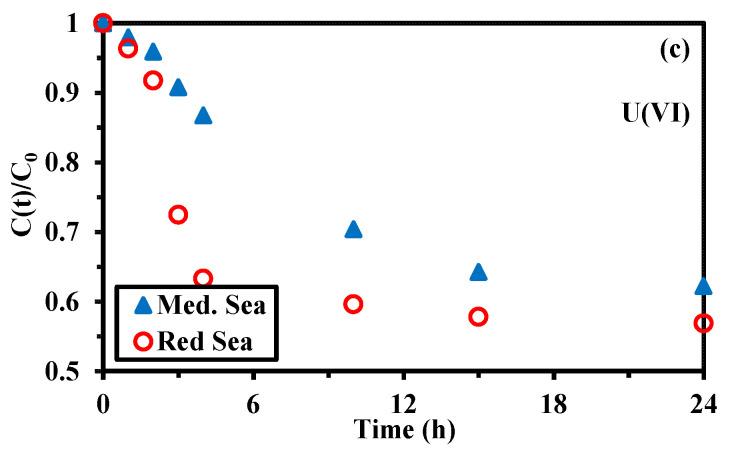
Uptake kinetics for the recovery of trace metal ions (Sr(II) (**a**), B(III) (**b**), and U(VI) (**c**)) from seawater samples (natural pH: ≈7.5; SD: 0.2 g L^−1^; v: 210 rpm).

Figure A20 reports the uptake kinetics for Sr(II) using SA*PEI sorbent for both the Mediterranean and Red Sea water samples. The complexity of the solutions strongly decreases the mass transfer properties: (a) for Sr(II) and B(III) a short lag phase lasting for 2–3 h occurs before the concentrations decrease (not observed with uranyl), and (b) reaching the equilibrium requires about 24 h of contact. Bezhin et al. [66] compared different sorbents for the recovery of Cs(I) and Sr(II) from seawater; they also reported quite slow kinetics (in most cases equilibrium was reached after more than 24 h of agitation, especially for strontium removal); the faster Sr(II) uptake was reported for copper potassium ferrocyanide supported on phosphorylated wood. In the case of Sr(II) sorption from seawater using alginate microspheres, Hong et al. observed also slow kinetics: 6 h are necessary (with high SD: 2 g L^−1^; herein the SD: 0.2 g L^−1^).

**Figure A21:**
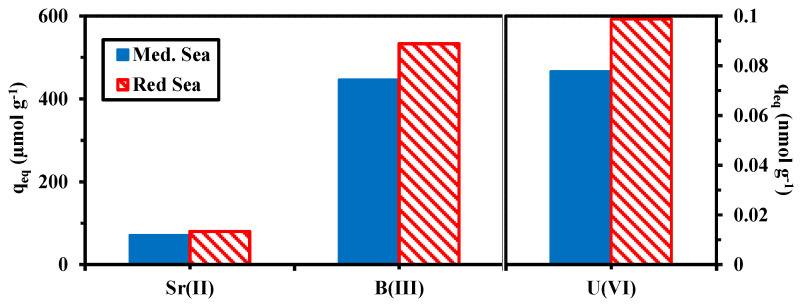
Recovery of trace metal ions (Sr(II), B(III), and U(VI)) from seawater samples (natural pH: ≈7.5; SD: 0.2 g L^−1^; time: 24 h; v: 210 rpm).

The sorption capacities reached under selected experimental conditions (which do not cover the saturation of the sorbent) are reported in Figure A21. The direct comparison of sorption capacities in seawater with the values reached with synthetic solutions is not possible; the pH is not adjusted (the “natural” pH is close to 7.5; pH_eq_ ≈7.6); and the sorbent dosages are not the same. However, in synthetic solution, for Sr(II) residual concentrations in the range 2.41–2.76 mg Sr L^−1^ the sorption capacities onto SA*PEI at pH 5.19 range between 2.90 and 4.37 mg Sr g^−1^ (i.e., 33–50 µmol Sr g^−1^; SD: 0.67 g L^−1^). In the case of seawater samples, for residual concentrations ranging between 2.99 and 4.57 mg Sr L^−1^, the sorption capacities reach 6.14–6.99 mg Sr g^−1^ (i.e., 70–80 µmol Sr g^−1^, SD: 0.2 g L^−1^). This means that despite the complexity of the background solution, which affects strontium speciation, the sorption is weakly affected. Kirishima et al. [82] discussed the speciation of strontium in seawater; they concluded that the metal may coexist under two forms sulfate “aquo” complex (i.e., SrSO_4aq_, ≈60%) and free Sr^2+^ (≈40%). Bezhin et al. [66] summarized the maximum sorption capacities of three sorbents for Sr(II) removal from seawater. In the case of nanostructured crystalline hydroxyapatite, they found a capacity close to 0.21 mg Sr g^−1^; for biogenic amorphous hydroxyapatite they cite a capacity close to 2.3 mg Sr g^−1^. The maximum sorption capacity reaches about 7 mg Sr g^−1^ for birnessite-type sorbent (i.e., a layered manganese oxide conditioned under sodium form). Therefore, despite the unsaturation of SA*PEI under selected experimental conditions, the sorption capacities sound very attractive and competitive.

Figure A21 also shows the data for boron and uranyl ions (at trace levels, apart from major metals). The sorption levels for boron range between 4.8 and 5.8 mg B g^−1^ (i.e., 446–533 µmol B g^−1^) are higher than those reached for uranyl (i.e., 0.078–0.098 µmol g^−1^). The weak sorption of uranyl may be explained by the low concentration of uranium in seawater but also perhaps by the effect of metal speciation under complex composition. Indeed, Amphlett et al. [83] showed that in seawater uranyl ions are mainly present as anionic species (more specifically: UO_2_(SO_4_)_3_^4−^), which may be poorly available for binding onto sulfonic groups. The distribution ratios for Sr(II), B(III), and U(VI) are close to D: 1.53–2.05, 1.66–1.94, and 3.04–3.79 L g^−1^, respectively (i.e., much higher than the D values obtained for major elements, below 0.06 L g^−1^). The drastic differences in the relative concentrations of major and trace elements strongly affect the distribution ratios and then the significance of selectivity coefficients (SC_Sr/metal_). However, a qualitative ranking can be evaluated:

Na(I) (72–86) > Mg(II) (48–66) > Ca(II) (34–43) > K(I) (26–38) >> B(III) (0.79–1.23) > U(VI) (0.40–0.68).

This ranking is roughly consistent with the concentrating effect (CF = q_eq_/C_0_, L g^−1^):

Na(I) (≈0.023) < Mg(II) (≈0.031) < Ca(II) (≈0.045) < K(I) (≈0.056) << Sr(II) (≈1.31) ≈ B(III) (≈1.32) < U(VI) (≈2.02).

The distribution ratio (i.e., D) increases with the pH (not shown). For Na(I), K(I), Mg(II) and Ca(II), D varies between 0.02 and 0.06 L g^−1^, this is 2 orders of magnitude lower than the D ratio for Sr(II) (i.e., 1.53), B(III) (i.e., 1.94) and U(VI) (i.e., 3.79). This comparison shows the preference of SA*PEI for Sr(II) (and boron or uranium) against alkaline and alkaline-earth metals.

These preliminary results demonstrate that SA*PEI maintains good sorption performance, concentrating effect for Sr(II) in seawater, despite the complexity of the matrix. Noteworthy, the sorbent shows a remarkable concentrating effect for uranyl ions (despite the effect of metal speciation).

## 3. Materials and Methods

### 3.1. Materials

Brown algae (*Laminaria digitata*) was supplied by Setalg (Pleubian-France). Branched polyethylene imine (PEI; 50 %, *w*/*w* in water), cesium nitrate (CsNO_3_; 99%), strontium nitrate (Sr(NO_3_)_2_; 99.99 %), sodium hydroxide (NaOH: ≥97.0%), calcium chloride (CaCl_2_ ≥ 99.9%, in synthesis processes), 2-propene-1-sulfonic acid (≥99.99%), hydroxylamine-O-sulfonic acid (≥99.99%), potassium persulfate (99.99%), sodium metabisulfite (99.99%) and glutaraldehyde were purchased from Sigma-Aldrich (Taufkirchen, Germany). Poly(ethylene glycol diglycidyl ether) (PEGDGE) is used for enhancing the stability through crosslinking process. The salts used in selectivity tests (i.e., NaCl (≥99.98%), MgCl_2_·6H_2_O (99%), FeCl_3_ (≥99.5%), AlCl_3_·6H_2_O (95%)), were obtained from Guangdong Guanghua, Sci-Tech Co., Ltd. (Guangdong, China). Calcium chloride (CaCl_2_, 99.1%) was supplied from Shanghai Makclin Biochemical Co., Ltd. (Shanghai, China). All other reagents are Prolabo products, which were used as received.

### 3.2. Synthesis of Sorbents

**Scheme A1:**
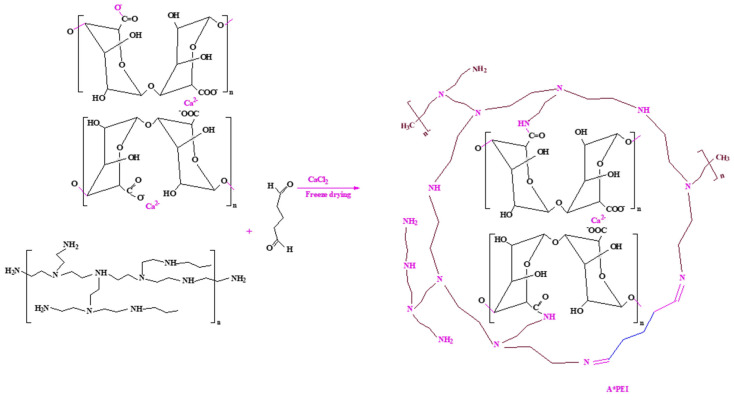
Synthesis and structure of A*PEI.

The synthesis of A*PEI has already been described. The concept is based on the partial thermal extraction of alginate from algal biomass using Na_2_CO_3_. In a second step, PEI was added to the mixture, which was further distributed through a thin nozzle into an ionotropic gelation solution (containing both CaCl_2_ and glutaraldehyde, for creating an interpenetrated network associating carboxylate gelation with calcium and crosslinking of amine groups with glutaraldehyde) (Scheme A1, see Appendix C, which reports the detailed experimental conditions).

**Scheme A2:**
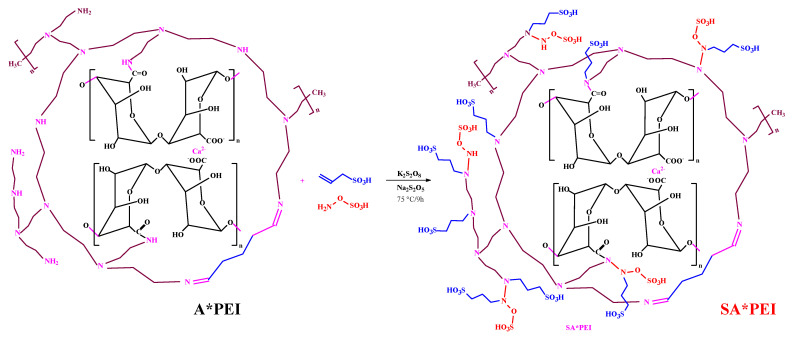
Synthesis and structure of SA*PEI.

The one-pot synthesis of SA*PEI (4 g) consisted of an original reaction involving the reaction of two reagents bearing sulfonic acid groups (i.e., 2-propene-1-sulfonic acid (1.0 g) and hydroxylamine-o-sulfonic acid (0.8 g)) with amine groups from PEI. The reaction took place under reflux (at 90 °C), to produce sulfonic acid derivative (5.64 g; yield: ≈97% on mass balance). Appendix C (and Scheme A2) provides detailed experimental conditions. Scheme 2 shows the expected structure of the sorbent.

### 3.3. Characterization of Sorbents

The pH of zero charge (pHpzc) was measured using the pH-drift method [84]. The FTIR spectra of samples (incorporated into a KBr disk, after dryness at 60 °C) were collected using an IRTracer-100 FT-IR spectrometer (Shimadzu, Tokyo, Japan). SEM analyses were acquired using a Phenom ProX -SEM; Thermo Fisher Scientific (Eindhoven, The Netherlands), while the chemical composition of the sorbents (raw beads, after functionalization and after metal-loading) was investigated using EDX analysis (energy dispersive X-ray analysis, coupled to the SEM system). The BET surface area and the porosity of the sorbents were determined using adsorption and desorption branches of N_2_ isotherms through Micromeritics TriStar- II; Norcross, GA, USA, (system-77 K), while the BET equation was used for surface area determination and the BJH method for porosity characterization. The samples were firstly swept under N2 gas atmosphere for 4 h at 120 °C before testing. The thermal decomposition (thermogravimetric analysis; TGA) of the samples was determined using TG-DTA (Netzsch STA: 449-F3 Jupiter, NETZSCH-Gerätebau HGmbh, Selb, Germany); the analysis was performed under nitrogen atmosphere, with 10 °C min-1 temperature ramp. The pH of the solution was adjusted using a S220 Seven Compact pH/ionometer (Mettler-Toledo, Shanghai, China). Strontium (and other metal ions) concentration were measured using inductively coupled plasma atomic emission spectrometer- ICP-AES (ICPS-7510 Shimadzu, Tokyo, Japan) (after filtration using filter membranes, 1.2 μm pore size). Sodium concentration in the solution was analyzed by flame atomic absorption spectrophotometry (FAAS-AA 7000, Shimadzu, Tokyo, Japan).

### 3.4. Sorption and Desorption Procedures

Sorption and desorption tests were performed in batches. The suspensions containing a given solid/liquid ratio (sorbent dose, SD = m/L, with m mass of sorbent (g) and V volume of solution (L), at concentration C_0_, mmol Sr L^−1^) were agitated at 210 rpm velocity. Unless specified, the standard temperature was 21 ± 1 °C. For uptake kinetics, homogeneous samples were collected at fixed contact times and the residual concentration (C_eq_ or C_(t)_, mmol Sr L^−1^) was determined by ICP-AES. For sorption isotherms, the contact time was fixed to 48 h. The initial pH_0_ of the solution was fixed using 0.1/1 M NaOH or HNO_3_ solutions; the pH was not controlled during sorption, but the equilibrium pH_eq_ was monitored. The sorption capacity q_eq_ (mmol Sr g^−1^) was deduced from the mass balance equation; q_eq_ = (C_0_ − C_eq_) × V/m. The same method was used for investigating the sorption properties from multi-component equimolar solutions, and for the evaluation of sorption properties from seawater samples (two samples collected from the Mediterranean Sea and the Red Sea).

For the study of desorption properties, the same experimental procedure was used (batch tests); the Sr-loaded sorbents were collected from uptake kinetics. The desorption kinetics using 0.3 M HCl solution were collected. In addition, the recycling of the sorbent was also tested for five successive cycles; a rinsing step (using demineralized water) was systematically processed between each step. By mass balance, it was possible calculating the sorption and desorption efficiencies.

Detailed experimental conditions are directly reported in the caption of the Figures.

## 4. Conclusions

The successful grafting of two types of sulfonic groups onto A*PEI using simultaneously two sulfonating agents (2-propylene-1-sulfonic acid and hydroxylamine-O-sulfonic acid) triples the maximum sorption capacity (q_eq,exp_) of SA*PEI for Sr(II) from 0.61 to 1.91 mmol Sr g^−1^, at pH_eq_ ≈4.5–5. S content reaches about 1 mmol S g^−1^ (maintaining N content close to 2.14 mmol N g^−1^). The optimum initial pH is strictly found at pH_0_ 4 for sulfonated sorbent (at pH_0_ 5 for A*PEI). Sorption isotherms for SA*PEI can be fitted by the Sips and the Langmuir dual site equations (confirmation of the contribution of both sulfonate groups and amine—or carboxyl—groups, consistently with FTIR characterization). The sorption onto SA*PEI is endothermic (shown by the substantial increase in sorption capacity and affinity coefficient with temperature increase from 20 to 50 °C). The sulfonation also improves mass transfer properties: Equilibrium is reached within 40 min (vs. 120 min for pristine beads), consistently with the enhancement of porous properties; the kinetic profiles are fitted by the pseudo-first-order rate equation. The effective diffusivity coefficient (D_e_) is about one order of magnitude lower than the self-diffusivity of strontium in water (limited contribution of intraparticle diffusion in the kinetic).

Sorption in multi-component (equimolar) solutions show the marked preference of SA*PEI for Sr(II) (and Cs(I), to a lesser extent) against alkaline, alkaline-earth, and heavy metals, especially at pH_eq_ close to 4. Quantitative structure-activity relationship tools confirm that strontium and cesium keep out the trends followed by other competitor metals. For these competitor metals, the distribution ratio can be relatively well correlated with the covalent index (rather than the ionic index). Selectivity for Sr(II) increases with increasing the absolute value of hydration enthalpy and decreasing the ionic radius and the atomic number (in each group of mono-, di-, and tri-valent cations). In even more complex solutions (i.e., seawater), SA*PEI keeps goods affinity for Sr(II).

Strontium can be readily (and fastly) desorbed from loaded sorbent using 0.3 M HCl solution as the eluent. Total desorption allows re-using the sorbent for successive cycles. The sulfonation notably increased the stability in sorption performance for sulfonated sorbent (loss in sorption efficiency does not exceed 1.7%) at the fifth cycle, compared with A*PEI (loss close to 12%).

## Figures and Tables

**Table 1:** Sr(II) uptake kinetics using A*PEI and SA*APEI—parameters of PFORE, PSORE, and RIDE models.

Sorbent			A*PEI	SA*PEI	
Model	Parameter	Temperature	T: 20 ± 1 °C	T: 20 ± 1 °C	T: 50 ± 1 °C
		Unit			
Experimental	q_eq_	mmol Sr g^−1^	0.179	0.926	1.17
PFORE	q_eq,1_	mmol Sr g^−1^	0.198	0.974	1.23
	k_1_ × 10^2^	min^−1^	2.04	5.01	5.80
	R^2^	-	0.969	0.976	0.977
	AIC	-	−144	−96	−91
PSORE	q_eq,2_	mmol Sr g^−1^	0.268	1.15	1.43
	k_2_ × 10^2^	g mmol^−1^ min^−1^	26.8	4.93	4.85
	R^2^	-	0.952	0.938	0.937
	AIC	-	−139	−83	−77
RIDE	D_e_ × 10^9^	m^2^ min^−1^	6.86	7.13	6.36
	R^2^	-	0.944	0.940	0.936
	AIC	-	−133	−82	−75

**Table 2:** Sr(II) sorption isotherms using A*PEI and SA*PEI—parameters of Langmuir, Freundlich, Sips, and Temkin models.

Sorbent		A*PEI	SA*PEI	
Temperature		20 ± 1 °C	20 ± 1 °C	50 ± 1 °C
Model	Parameter			
Experim.	q_m,exp._	0.607	1.91	2.40
Langmuir	q_eq,L_	0.977	2.05	2.33
	b_L_	0.278	1.94	4.88
	R^2^	0.981	0.992	0.969
	AIC	−214	−164	−110
Langmuir dual site	q_eq,L1_	-	0.192	0.903
	b_L1_	-	63.4	34.0
	q_eq,L2_	-	1.94	1.65
	b_L2_	-	1.41	1.36
	R^2^	-	0.994	0.986
	AIC	-	−180	−136
Freundlich	k_F_	0.221	1.18	1.66
	n_F_	1.67	2.87	3.61
	R^2^	0.986	0.968	0.956
	AIC	−231	−128	−104
Sips	q_eq,S_	-	2.33	2.83
	b_S_	-	1.29	1.83
	n_S_	-	1.29	1.62
	R^2^	-	0.994	0.984
	AIC	-	−177	−134
Temkin	A_T_	9.37	46.4	162
	b_T_	18,440	7028	7552
	R^2^	0.899	0.981	0.984
	AIC	−168	−145	−118
Temkin-II	q_T_	-	0.434	0.395
	K_T_	-	18.2	92.2
	R^2^	-	0.991	0.984
	AIC	-	−168	−137

Units: q, mmol g^−1^; b and K_T_, L mmol^−1^; n, dimensionless; k_F_, mmol^1−1/nF^ L^−1/nF^ g^−1^; A_T_, L mmol^−1^; b_T_, J mmol^−1^; fitting processed on cumulative triplicates.

**Table 3:** Sorbent recycling—Sorption efficiency (SE, %) and desorption efficiency (DE, %) for five successive cycles.

Sorbent	A*PEI				SA*PEI			
Cycle	SE (%)		DE (%)		SE (%)		DE (%)	
	Aver.	St. dev.	Aver.	St. dev.	Aver.	St. dev.	Aver.	St. dev.
#1	10.73	0.85	100.5	0.4	58.7	1.8	99.9	0.2
#2	10.22	0.85	104.9	7.6	58.6	1.8	100.0	0.1
#3	9.86	0.61	100.1	0.6	58.3	1.7	100.0	0.1
#4	9.75	0.65	99.7	0.1	57.8	1.8	100.0	0.0
#5	9.46	1.01	100.2	0.3	57.7	1.8	100.0	0.0
Loss (5th/1st)	11.8%		S and C		1.7%		S and C	

## Data Availability

Data can be obtained from the authors on demand.

## Appendix A. Characterization of Materials

### Appendix A.2. Textural Properties

The isotherms of adsorption and desorption of N_2_ are characterized by a Type II profile according to IUPAC classification [85]: The first section corresponds to a concave shape followed by a linear section and terminated with a convex section (Figure A3a). This type of isotherm is usually associated with non-porous or macroporous materials. However, substantial differences are observed between A*PEI and its sulfonated derivative (SA*PEI). First, the specific surface area (SSA) determined by BET analysis is substantially increased from 2.1 to 7.4 m^2^ g^−1^. In addition, the hysteresis loop (sorption vs. desorption branches) is much less marked for raw sorbent than its derivative that exhibits Type H3 hysteresis: A*PEI profile correlates with the so-called Type II(a) isotherm, while SA*PEI corresponds more to Type II(b) isotherm. H3 hysteresis loops are usually associated with aggregates of platy particles [86].

In the current case, the sorbent is based on a hydrogel structure resulting from the interaction of alginate fraction from algal biomass and polyethylenimine; the sulfonation of amine groups involves an expansion of the polymer structure with increased flexibility (which may explain the larger hysteresis loop). The porous volume increases tenfold after sulfonation; this is consistent with the larger SSA. Figure A3b shows the distribution of pore width for the two sorbents. Not considering the broad section of the curves that correspond to the macroporous regions (above 500 Å), A*PEI shows a principal pore size fraction (which maximum is close to 34 Å); the pore width is calculated (BJH method) close to 99–94 Å (restricted variation between measurements from adsorption and desorption branches). In the case of sulfonated sorbent, a broader maximum is observed around 214 Å; the difference in the values obtained from adsorption and desorption branches (332–199 Å) is substantially larger than for A*PEI. From these observations, it is possible anticipating that SA*PEI may have more favorable mass transfer characteristics than A*PEI.

### Appendix A.3. Thermal Degradation Properties

The investigation of the thermal degradation of sorbents is reported in TGA and DrTG profiles (Figure 1); Figure A4 shows the inset and outset temperatures with detailed weight losses (WLs) for TGA curve. In the TGA curves, three transitions can be identified for A*PEI against four transitions in SA*PEI.

The first transition corresponds to water release from sorbent at temperature below 177–211 °C, representing 20.2% and 10.8% WLs for A*PEI and SA*PEI, respectively.

In the second stage of the degradation, the WLs represent 46.9% (177–404 °C) and 31.0% (211–389 °C), respectively. This step may be associated with the depolymerization of PEI and carbohydrate ring and the degradation of carboxyl groups.

The last transition for A*PEI (404–890 °C) leads to a complementary WL of 26%. This phase corresponds to the degradation of PEI (into CO, CO_2_, and CH_4_) [87], and the final degradation of the char (produced in the preceding step).

This degradation profile is roughly consistent with the profile reported by Godiya et al. [88]; however, the curve is shifted toward higher temperatures of degradation.

Contrary to A*PEI, the end of the degradation profile of SA*PEI shows two successive waves: (a) 389–604 °C (WL: 27%), and (b) 604–890 °C (WL: 14%). The first transition corresponds to sulfonate degradation (reported above 320 for sulfonate polystyrene/silica composite, [89]). The last section corresponds to the degradation of the char. The sulfonate derivative increases the thermal resistance of the composite. The residue represents 17.2% while for the precursor the residue was less than 7%.

DrTG curves confirm these trends (Figure 1b) and the global higher stability of the sulfonated derivative. The main observations can be summarized: Hardly marked inflection at the water release phase, shift from 293.2 to 308.4 °C for the second step in the degradation, the shift of the peak at 498.4 °C (in A*PEI) toward lower temperature with functionalization to 468.8 °C, accompanied by a shoulder at 537 °C, and shift of sharp weakly intense peak at 580.85 °C, which is shifted toward higher temperature (i.e., 681.1 °C).

### Appendix A.4. FTIR Spectroscopy

The sorbents are constituted of algal biomass (including alginate, meaning carboxylic acid), polyethyleneimine (PEI, meaning primary, secondary, and tertiary amines), and sulfonic acid (after functionalization) (Figure 2). The proper complexity of algal biomass that contains many constituents [90,91], such as proteins, minerals, and carbohydrates (alginate, mannitol, laminarin, cellulose, or polyphenols) makes the interpretation of FTIR spectra very complex.

Rodriguez et al. [91] identified alginate in brown algae through: Guluronic acid residues with bands at 1290, 1080, 1025, and 787 cm^−1^, mannuronic acid residues at 1320, 1030, 1019, 878, and 808 cm^−1^, and the ν(C-O) of uronic acids at 948 cm^−1^. The sulfate esters of fucoidan (representing a weak fraction of carbohydrates) may be identified by bands at 1260–1195 cm^−1^ (ν(S=O)). In the case of the interactions of PEI with Mesquite Gum (MG, which also bears carboxylic groups), Pinilla-Torres et al. [92] reported a series of bands representing the typical bands associated with PEI and carboxylic functions from the MG:

- 3420 cm^−1^: ν(N-H) primary amine
- 3280 cm^−1^: ν(N-H), primary amine and Amide A band
- 1648 cm^−1^: ν(C=O)/ν(C-N), Amide I band
- 1551 cm^−1^: ν(N-H)_primary_ in-plane δ(N-H)/ν(C-N)/ν(C-C), Amide II band
- 1241 cm^−1^: ν(C-N)/δ(N-H), Amide III band
- 1074 cm^−1^: ν(C-N)

Table A1 summarizes the main peaks appearing on the spectra of the sorbents before and after Sr(II) sorption, and after the fifth cycle of desorption (to evaluate the potential degradation of the sorbents). The sorbents show a broad band at 3430–3420 cm^−1^, which is assigned to the overlapping of ν(N-H) and ν(O-H), while the bands at 2950–2930 cm^−1^ correspond to ν(C-H). These bands are not significantly influenced by A*PEI functionalization nor interactions with Sr(II) (metal sorption and desorption).

The main effects of sulfonation on the FTIR spectrum of A*PEI are identified in Figure A5 in the region of amine/amide bands, and carboxylic groups: The bands are shifted and/or width-changed. In addition, typical bands of sulfonic groups are identified at 1159 cm^−1^, for example.

**Table A1:** Assignment of main FTIR bands for A*PEI and SA*PEI before and after Sr(II) sorption, and after the fifth desorption (sorbent recycling).

Vibration	A*PEI	A*PEI + Sr(II)	A*PEI after 5th Desorption	SA*PEI	SA*PEI + Sr(II)	SA*PEI after 5th Desorption	Ref.
ν(N-H) + ν(O-H)	3429	3449–3428	3447–3429	3418	3443–3429	3422	[93]
ν(C-H) (aliphatic)	2934	2939	2934	2947	2932	2953	[93]
ν(C=O)	1767	1721	1765				[93]
δ(N-H)_prim._	1622	1634	1620	1632	1618	1622	[93]
O-H, δ(N-H)_2nd_, δ(C-H)	1383	1429	1422	1435	1429	1435	[93]
ν(S=O)						1339–1315	
ν(C-O)				1256		1256	
ν_s_(C-N), ν_as_(S=O)				1159	1171	1165	[94,95]
ν(C-O-C) carbohydr.	1092	1083	1086	1084	1086	1084	[96]
δ(O-H), ν_s_(S=O), ν_sk_(C-O), ν(C-N)	1034	1031	1031	1030	1032	1030	[95,96,97,98]
δ(O-H)		943	945	941		941	[93]
ν(S=O) and δ(C-H)	847, 876	818	816, 852, 876	818, 852	816, 851, 878	818, 854	[98,99]
Sr-N, Sr-O bonds, ν(S=O)				602	573	555	[99,100]

In Figure A6, the mains changes brought by Sr(II) sorption are identified. For A*PEI (Figure A6a), the most significant changes concern:

- the region 1750–1700 cm^−1^, assigned to ν(C=O) for carboxylic groups: Shift toward lower wavenumber and formation of a triplet of bands,
- the region 1660–1580 cm^−1^, assigned to amide bands (overlapped with amine groups): Stronger signal with reduced width,
- the intense and broad band, resulting from the overlapping of different vibrations (O-H, δ(N-H)_2nd_, δ(C-H)): Shift toward higher wavenumber and width reduction, and
- the region 950–800 cm^−1^, assigned to δ(O-H), δ(C-H) signals: Variations in the intensity of the relevant signal.

Different changes in FTIR spectra can be observed for SA*PEI (Figure A6b). The main differences are observed:

(a) the region at ≈1702 cm^−1^ (weak shoulder), associated with residual ν(C=O) of carboxylic groups, that disappears (or is shifted toward lower wavenumber around 1652 cm^−1^, where it contributed to the widening of the band at 1632 cm^−1^,
(b) the region at ≈1632 cm^−1^, attributed to δ(N-H) (associated with amide I band): Widening,
(c) the region at ≈1256 cm^−1^, assigned to ν(C-O): Intensity reduction,
(d) the band at 1159 cm^−1^, corresponding mainly to ν_as_(S=O): Shift toward higher wavenumber,
(e) the band at 602 cm^−1^, assigned to ν(S=O): Shifted toward lower wavenumber (and/or replace with a signal associated with Sr-N and Sr-O bond).

After regeneration (at the end of the fifth sorption/desorption cycle), the FTIR spectra maintain significant changes (Figure A7). Hence, for A*PEI, the most representative differences concern:

(a) the band at 1620 cm^−1^: Width reduction,
(b) the band at 1383 cm^−1^: Width reduction and shift toward higher wavenumber, and
(c) the region at 950–800 cm^−1^: (weak) intensity reductions and shifts of local peaks.

In the case of SA*PEI, the sorbent appears relatively well restored and the FTIR spectra are close between pristine sorbent and regenerated material:

(a) the shoulder at 1702 cm^−1^: Significantly reduced,
(b) the band at 1159 cm^−1^: Shift toward higher wavenumber (though less than after Sr(II) sorption, and
(c) the band at 602 cm^−1^ is again shifted toward the lower wavenumber (more extensive than after Sr(II) sorption).

The comparison of the spectra suggests that the sorption of Sr(II) onto A*PEI involves amine and carboxyl groups; in the case of SA*PEI, the modification of the chemical environment of sulfonic appearing in the FTIR spectrum means that sulfonate groups are the main binding functional groups (with the contribution of amine and carboxylic groups). The regeneration of the sorbents (desorption with 0.3 M HCl solution, after the fifth desorption step) does not completely restore the material in their neat form: The functional groups (amine/amide, carboxyl, sulfonate) are partially regenerated.

This final analysis depends on the FTIR spectra of the materials conditioned at the pH used for Sr(II) sorption (Figure A7) and analyzed after contact with 0.3 M HCl solution (with strontium) to isolate the relevant effects of sorbent conditioning and proper metal-sorbent interactions (and desorption). Globally, the contact of the sorbents with the metal-free solutions at the pH selected for strontium sorption does not show significant differences with pristine sorbents. Therefore, the changes in the spectra observed after Sr(II) binding can be specifically attributed to the interactions of the metal with identified functional groups.

The contact of A*PEI with 0.3 M HCl solution weakly affects the FTIR spectrum: The most significant changes concern the bands at 1383 cm^−1^ (δ(N-H)), 1632 cm^−1^ (δ(N-H)), and 1713 cm^−1^ (shifted from 1767 cm^−1^, ν(C=O)); the modification of these bands was also observed after Sr(II) sorption. These bands are affected by the protonation of reactive groups, and/or their interaction with Sr(II). It is noteworthy that the regeneration of the sorbent restores the band at 1765 cm^−1^ contrary to the 0.3 M HCl-conditioned sorbent (Figure A7). In the case of SA*PEI, some specific bands appear (or are shifted) after being in contact with 0.3 M HCl solution: The band at 1748 cm^−1^, at 1383 cm^−1^ (strong shift of the band at 1435 cm^−1^), and 1128 cm^−1^. The recycled sorbent (after 5 cycles) shows a spectrum globally closer to the pristine sorbent than the protonated sorbent collected after contact with 0.3 M HCl solution (Figure A8). In addition, the FTIR spectrum of SA*PEI is less affected by sorption and desorption cycles than A*PEI; consistently with higher stability of sorption performances (Table 3).

### Appendix A.5. Elemental Analysis and pH_PZC_

The elemental analysis confirms the successful sulfonation of raw material (1.04 mmol S g^−1^; consistent with the increase in O content); N content is not affected by chemical modification (≈2.14 mmol N g^−1^). The grafting of acid reactive groups (sulfonic acid) induces a strong decrease of the pH_PZC_ from 7.73–7.84 to 4.53–4.64. This is consistent with the well-known character of sulfonic-based resins as strong cation exchangers. The concentration of background salt (0.1 M vs. 1 M NaCl) hardly changes pH_PZC_ values (Figure A9).

**Table A2:** Elemental analysis of A*PEI and SA*APEI.

Sorbent	C (%)	H (%)	O (%)	O (mmol g^−1^)	N (%)	N (mmol g^−1^)	S (%)	S (mmol g^−1^)
A*PEI	35.97	11.95	36.13	22.58	3.02	2.156	0.19	0.0593
SA*PEI	34.84	13.84	43.61	27.26	2.97	2.121	3.32	1.036

## Appendix B. Sorption Properties

### Appendix B.1. Modeling of Uptake Kinetics and Sorption Isotherms

**Table A3:** (**a**). Reminder on equations used for modeling uptake kinetics; (**b**). Reminder on equations used for modeling sorption isotherms.

(a)			
Model	Equation	Parameters	Ref.
PFORE	$q\left(t\right)=q_{\mathrm{eq},1}\left(1-{\;e}^{-k_{1}t}\right)$	q_eq,1_ (mmol g^−1^): Sorption capacity at equilibrium k_1_ (min^−1^): Apparent rate constant of PFORE	[101]
PSORE	$q\left(t\right)=\frac{q_{\mathrm{eq},2}^{2}k_{2}t}{1+k_{2}q_{\mathrm{eq},2}t}$	q_eq,2_ (mmol g^−1^): Sorption capacity at equilibrium k_2_ (g mmol^−1^ min^−1^): Apparent rate constant of PSORE	[101]
RIDE	$\frac{q\left(t\right)}{q_{\mathrm{eq}}}=1-\sum_{n=1}^{\infty }\frac{6\alpha \left(\alpha +1\right)\exp\left(\frac{-D_{e}q_{n}^{2}}{r^{2}}t\right)}{9+9\alpha +q_{n}^{2}\alpha^{2}}$ With qn being the non-zero roots of $\tan q_{n}=\frac{3{\;q}_{n}}{3+{\;\alpha q}_{n}^{2}}$ and $\frac{m\;q}{{V\;C}_{0}}=\frac{1}{1+\;\alpha }$	D_e_ (m^2^ min^−1^): Effective diffusivity coefficient	[102]
**(b)**			
**Model**	**Equation**	**Parameters**	**Ref.**
Langmuir	$q_{\mathrm{eq}}=\frac{q_{m,L}C_{\mathrm{eq}}}{1+b_{L}C_{\mathrm{eq}}}$	q_m,L_ (mmol g^−1^): Sorption capacity at saturation of monolayer b_L_ (L mmol^−1^): Affinity coefficient	[103]
Freundlich	$q_{\mathrm{eq}}={\;k}_{F}C_{\mathrm{eq}}^{1/n_{F}}$	k_F_ (mmol g^−1^)/(mmol L^−1^)^n^_F_ and n_F_: Empirical parameters of Freundlich equation	[104]
Sips	$q_{\mathrm{eq}}=\frac{q_{m,S}b_{S}C_{\mathrm{eq}}^{1/n_{S}}}{1+b_{S}C_{\mathrm{eq}}^{1/n_{S}}}$	q_m,L_ (mmol g^−1^), b_S_ (mmol L^−1^)^n^_S_, and n_S_: Empirical parameters of Sips equation (based on Langmuir and Freundlich equations)	[105]
Temkin	$q_{\mathrm{eq}}=\frac{\mathrm{RT}}{b_{T}}\ln\left(A_{T}{\;C}_{\mathrm{eq}}\right)$	A_T_ (L mmol^−1^): equilibrium binding capacity; b_T_: Temkin constant related to sorption heat (J kg^−1^ mol^−2^)	[71,106]

(m (g): mass of sorbent; V (L): Volume of solution; C_0_ (mmol L^−1^): initial concentration of the solution).

Akaike Information Criterion, AIC [107]:

$$\mathrm{AIC}=N\ln\left(\frac{\sum_{i=0}^{N}{\left(y_{i,\exp.}-y_{i,\mathrm{model}}\right)}^{2}}{N}\right)+2N_{p}+\frac{2N_{p}\left(N_{p}+1\right)}{N-N_{p}-1}$$

where N is the number of experimental points, N_p_ the number of model parameters, y_i,exp._ and y_i,model_ the experimental and calculated values of the tested variable.

### Appendix B.2. Effect of pH on Sr(II) Sorption

The distribution ratio is defined, at equilibrium, as the ratio between sorption capacity and residual metal concentration in the solution (D = q_eq_/C_eq_, L g^−1^). Figure A12 shows the log_10_ plots of D vs. equilibrium pH. The slope of this plot may be used for evaluating the stoichiometry of proton exchange in ion-exchange processes (i.e., +2 for the exchange of divalent cation with protons, +1 for monovalent cation; in the case of strong acid resin, or +1 for weak acid resin) [109]. From Figure 3, it is possible anticipating substantial differences between A*PEI and SA*PEI. For the reference material, the distribution coefficient continuously increases, and the slope is close to 0.163. On the opposite hand, for SA*PEI, whatever the temperature, two linear segments can be identified (with a breakpoint close to pH 4): in acidic solutions, the slopes range between 0.39 and 0.43, while near neutral pH, the slopes evolve between −0.36 and −0.40. Despite the good quality of the fits (R^2^ values), the slopes are not consistent with the divalent charge of strontium. The contribution of several types of reactive groups and/or alternative binding mechanisms could explain the impossibility of appropriately fitting the slopes with the stoichiometric exchange ratio. Notably, the absolute values of the slopes of the ascending and descending segments are very close (+0.431/−0.401 at 50 °C, +0.390/−0.360 at 20 °C); meaning that the numbers of protons exchanged (bound/released) per strontium are the same on both side of the extrema.

### Appendix B.3. Uptake Kinetics

The calculated sorption capacities at equilibrium slightly overestimate experimental values:

For A*PEI: 0.198 ± 0.003 mmol g^−1^ vs. 0.179 ± 0.010 mmol g^−1^.

For SA*PEI: 0.974 ± 0.009 mmol g^−1^ vs. 0.926 ± 0.002 mmol g^−1^.

The increase in temperature slightly increases the apparent rate coefficient:

A*PEI at 20 °C (5.02 ± 0.23 × 10^−2^ min^−1^) < SA*PEI at 50 °C (5.80 ± 0.07 × 10^−2^ min^−1^).

The fitted sorption capacities also slightly overestimate experimental values when varying temperature:

For SA*PEI at 20 °C: 0.974 ± 0.009 mmol g^−1^ vs. 0.926 ± 0.002 mmol g^−1^.

For SA*PEI at 20 °C: 1.227 ± 0.034 mmol g^−1^ vs. 1.170 ± 0.029 mmol g^−1^.

**Table A4:** Sr(II) uptake kinetics using A*PEI and SA*APEI—Parameters of PFORE, PSORE, and RIDE models (individual replicates).

Sorbent		A*PEI			SA*PEI					
Temperature		T: 20 ± 1 °C			T: 20 ± 1 °C			T: 50 ± 1 °C		
Model	Parameter	# 1	# 2	# 3	# 1	# 2	# 3	# 1	# 2	# 3
Experim.	q_eq_	0.181	0.177	0.180	0.939	0.921	0.917	1.18	1.20	1.13
PFORE	q_eq,1_	0.194	0.200	0.199	0.986	0.968	0.967	1.24	1.26	1.18
	k_1_ × 10^2^	2.20	1.89	2.06	5.14	5.21	4.70	5.72	5.88	5.80
	R^2^	0.976	0.949	0.971	0.975	0.974	0.973	0.977	0.976	0.977
	AIC	−151	−137	−149	−97	−97	−96	−92	−92	−92
PSORE	q_eq,2_	0.257	0.277	0.268	1.16	1.14	1.15	1.44	1.46	1.38
	k_2_ × 10^2^	7.43	5.40	6.45	5.03	5.22	4.56	4.72	4.83	5.01
	R^2^	0.962	0.929	0.955	0.936	0.935	0.937	0.937	0.937	0.937
	AIC	−146	−134	−145	−86	−86	−86	−80	−80	−80
RIDE	D_e_ × 10^9^	7.06	6.54	6.93	8.30	8.54	7.48	7.22	7.14	7.26
	R^2^	0.956	0.920	0.947	0.939	0.939	0.938	0.936	0.935	0.936
	AIC	−140	−129	−138	−81	−82	−81	−75	−74	−75

### Appendix B.4. Sorption Isotherms

**Table A5:** (**a**). Sr(II) sorption isotherms using A*PEI (individual replicates); (**b**). Sr(II) sorption isotherms using SA*PEI, at T: 20 °C (individual replicates); (**c**). Sr(II) sorption isotherms using SA*PEI, at T: 50 °C (individual replicates).

(a)				
		Series #		
Model	Parameter	1	2	3
Experim.	q_m,exp._	0.584	0.570	0.607
Langmuir	q_eq,L_	1.140	0.864	0.988
	b_L_	0.195	0.366	0.286
	R^2^	0.987	0.993	0.983
	AIC	−75	−83	−74
Freundlich	k_F_	0.199	0.233	0.230
	n_F_	1.54	1.77	1.70
	R^2^	0.991	0.993	0.991
	AIC	−82	−85	−81
Temkin	A_T_	8.32	9.51	10.4
	b_T_	18388	18348	18550
	R^2^	0.893	0.943	0.908
	AIC	−54	−62	−56
**(b)**				
		**Series #**		
**Model**	**Parameter**	**1**	**2**	**3**
Experim.	q_m,exp._	1.86	1.84	1.91
Langmuir	q_eq,L_	2.02	2.02	2.13
	b_L_	1.93	2.15	1.75
	R^2^	0.993	0.995	0.994
	AIC	−55	−58	−57
Freundlich	k_F_	2.90	1.20	1.19
	n_F_	1.67	2.92	2.77
	R^2^	0.908	0.971	0.975
	AIC	28	−42	−43
Sips	q_eq,S_	2.34	2.24	2.41
	b_S_	1.21	1.47	1.21
	n_S_	1.34	1.26	1.27
	R^2^	0.995	0.996	0.995
	AIC	−58	−61	−58
Temkin	A_T_	47.1	49.9	42.3
	b_T_	7187	7114	6785
	R^2^	0.985	0.988	0.982
	AIC	−49	−51	−47
**(c)**				
		**Series #**		
**Model**	**Parameter**	**1**	**2**	**3**
Experim.	q_m,exp._	2.40	2.28	2.34
Langmuir	q_eq,L_	2.38	2.27	2.33
	b_L_	4.90	4.68	4.70
	R^2^	0.968	0.969	0.988
	AIC	−33	−34	−46
Freundlich	k_F_	1.70	1.63	1.66
	n_F_	3.66	3.64	3.55
	R^2^	0.963	0.968	0.960
	AIC	−33	−36	−33
Sips	q_eq,S_	2.97	2.96	2.61
	b_S_	1.69	1.48	2.51
	n_S_	1.69	1.80	1.37
	R^2^	0.985	0.986	0.995
	AIC	−40	−42	−52
Temkin	A_T_	165.7	205.3	121.8
	b_T_	7404	8081	7144
	R^2^	0.987	0.985	0.995
	AIC	−45	−45	−56

Units: q, mmol g^−1^; b, L mmol^−1^; n, dimensionless; k_F_, mmol^1−1/nF^ L^−1/nF^ g^−1^; A_T_, L mmol^−1^;b_T_, J mmol^−1^.

**Table A6:** Comparison of Sr(II) sorption properties with alternative sorbents (for T ≈ 20 °C; Nat.: natural pH or non-documented).

Sorbent	pH	Time (min)	q_m,exp_ (mmol g^−1^)	q_m,L_ (mmol g^−1^)	b_L_ (L mmol^−1^)	Ref.
Dowex 50W8 sulfonic resin	3.7	60	1.43	1.43	206	[36]
Alginate microsphere	6	1440	1.20	1.27	8.24	[58]
Resorcinol-formaldehyde resin	7	1440	-	1.14	-	[34]
Sulfonated polyaniline sorbent	Nat.	40	1.01	1.05	4.73	[110]
Amidoximated algal/PEI beads	6	90	2.16	2.36	2.01	[38]
Crab carapace	Nat.	240	0.038	0.045	11.4	[111]
SrTreat^®^	Nat.	60	0.104	0.109	265	[111]
Kurion-TS™	Nat.	60	0.128	0.230	492	[111]
Mixed-bed resin (T-46/A-33)	7	30	-	0.109	0.084	[35]
Functionalized silica beads	8	60	1.38	1.57	1.41	[74]
Magnetic composite sulfonated sorbent	10	180	0.539	-	-	[30]
*Salvadora persica* biomass	7	60	-	0.474	0.237	[27]
Fly ash-based zeolite	5.4	720	0.681	0.749	0.756	[112]
*Photinia serrulata* leaf	>4	30	0.120	0.138	11.0	[113]
*S. cerevisiae*-Fe_3_O_4_ composite	6	960	-	0.234	1.33	[29]
*Bacillus pumilus* SWU7–1	7	7200	3.14	3.42	2.89	[28]
Modified montmorillonite	7	30	-	0.028	40.2	[114]
Granular manganese oxide	-	4800	1.2	1.9	0.1	[73]
Zr-metal-organic framework	Nat.	5	-	0.871	2.51	[115]
Functionalized graphene oxide	2	60	1.37	1.44	3.03	[75]
SLS/polyacrylonitrile	11.5	800	0.4	0.376	10.8	[64]
PVA/graphene oxide aerogel	7	480	0.228	0.229	239	[116]
Graphene oxide	5	20	0.97	1.50	0.850	[76]
ZrSn(IV) phosphate nanocomp.	8	120	-	0.202	3.94	[117]
A*PEI	5	120	0.607	0.977	0.278	*This study*
SA*PEI	5	40	1.91	2.05	1.94	*This study*

### Appendix B.5. Sorption Selectivity

The distribution ratios (i.e., D, L g^−1^, not shown) increase with pH for Na(I), contrary to Sr(II) (which showed a maximum for pH 4), and decrease with increasing Na,Cl concentration (from 0.0093 to 0.0029 L g^−1^). At pH 4, the D ratio for Sr(II) is 215 to 465 times higher than the value for Na(I). This is consistent with competitor studies (Section 2.2.5 and Section 2.2.6) and with the remarkable preference of SA*PEI for Sr(II) against Na(I); this is a good indication of the interest of the sorbent for application in seawater (see Section 2.3).

### Appendix B.7. Application to Seawater Samples

The Red Sea is characterized by a higher Sr(II) content than the Mediterranean sea (Table A6). Actually, this may be explained by the geologic environment of the local Egyptian coast. Strontium deposits occur in the Quseir-Marsa Alam area in the sedimentary rocks of the late Miocene. The concentration of strontium reaches up to 1% (as SrSO_4_ mineral). The Quseir-Marsa Alam area is regularly exposed to violent and short rains during the winter season, forming torrents that lead to the transfer of many blocks and/or fragments of rocks (containing SrSO_4_) to the Red Sea, in addition to natural drainage of strontium-containing solutions [118,119].

**Table A7:** Composition of seawater samples (major metal ions and selected traces).

Metal Ion	Units	Mediterranean Sea	Red Sea
Na(I)	g L^−1^	13.03	14.10
K(I)	mg L^−1^	554.8	489.5
Mg(II)	mg L^−1^	1424	1504
Ca(II)	mg L^−1^	555.3	568.4
Sr(II)	mg L^−1^	4.218	5.968
B(III)	mg L^−1^	3.856	4.119
U(VI)	µg L^−1^	9.8	10.9

## Appendix C. Synthesis of Sorbents

### Appendix C.1. Manufacturing of A*PEI Beads

The synthesis of algal-PEI beads was already described [120]. Algal/PEI beads were prepared through three steps (see Scheme A1). After grinding (≈250 µm) and sieving the algal biomass (*L. digitata*), the algae (30 g) was dispersed into 800 mL of Na_2_CO_3_ solution (1%, *w/w*), this suspension was maintained under agitation at 50 °C for 24 h. This step allows the partial extraction of alginate from algae. Five mL of PEI (50%, *w/w*) was added to the mixture (second step) before dropping the mixture into 2 L of CaCl_2_ solution (1%, *w/w*) for ionotropic gelation of extracted alginate (third step) (by the interaction of calcium with carboxylate groups). This gelation bath was completed with glutaraldehyde (GA, 5 mL, 50% *w/w*) for linkage between amine groups in PEI and for creating an interpenetrating network (alginate carboxylic groups/Ca(II) and PE amine groups/GA). These reactions contribute to the stability of the material. The beads were freeze-dried for two days (−52 °C, 0.1 mbar) for work organization reasons; however, alternative tests showed that shorter operating time (i.e., overnight, ≈15 h) is sufficient for achieving the freeze-drying. Scheme A1 reports the structure and the principle of A*PEI synthesis.

### Appendix C.2. Functionalization of A*PEI (Synthesis of SA*PEI)

The one-pot sulfonation of A*PEI was processed in a closed reactor (adapted for processing redox reactions). Four g of dry A*PEI beads were dispersed into demineralized water (20 mL) in the reactor. Potassium persulfate (0.2 g) and sodium metasulfite (0.1 g) were added (and dissolved in the aqueous suspension). The sulfonating agents (i.e., 2-propene-1-sulfonic acid (1.0 g) and hydroxylamine-O-sulfonic acid (0.8 g)) were first dissolved in water (10 mL), before adding dropwise into the reactor. The suspension was refluxed (at 90 °C) for 9 h. The beads were filtered off, washed with water, and acetone before being dried at 50 °C for 12 h. Persulfate and bisulfide redox initiators orientate the bonding of C=C with >NH and –NH_2_ groups [121]. The synthesis is summarized in Scheme A2.
